# Role prompting modulates linguistic style but not clinical decision structure in GPT-5 tumour board simulation

**DOI:** 10.1038/s41746-026-03069-4

**Published:** 2026-08-13

**Authors:** Derna Stifini, Andrea Della Penna, André L. Mihaljevic, Michael Bitzer, Carsten Eickhoff, Ivan Capobianco

**Affiliations:** 1 Department of General, Visceral and Transplant Surgery, University Hospital Tübingen, Tübingen, Germany; 2 Department of Internal Medicine I, University Hospital Tübingen, Tübingen, Germany; 3 Center for Digital Health, University Hospital Tübingen, Tübingen, Germany

**Keywords:** Cancer, Computational biology and bioinformatics, Oncology

## Abstract

Multidisciplinary tumour boards (MDTs) are the standard for gastrointestinal oncological decision-making but remain resource-intensive. Whether specialty-specific role prompting induces genuinely distinct clinical reasoning in large language models (LLMs)—or merely role-appropriate language around an invariant output—has not been systematically tested. We applied five zero-shot prompting frameworks and a majority-vote ensemble to GPT-5 across 100 gastrointestinal oncology cases with MDT-validated decisions: a simulated MDT, multi-expert deliberation, three specialist personas, and a majority-vote ensemble. Concordance with MDT recommendations ranged from 78% to 87%, with no significant inter-framework differences (Cochran’s *Q* = 8.46, *p* = 0.133). Specialty-characteristic language was near-universal (97–100%) but uncorrelated with accuracy. Embedding analysis revealed high semantic similarity across personas (cosine similarity 0.805–0.836; η² = 0.049), contrasting with substantially greater output separation under multi-expert deliberation (η² = 0.554–0.581). GPT-5 reliably adapts linguistic style to clinical personas but produces limited specialty-specific output diversity, supporting its role as a decision-support adjunct rather than an autonomous specialist simulator.

## Introduction

Multidisciplinary tumour boards (MDTs) are the established standard for complex oncological decision-making, with meta-analyses demonstrating improved 5-year survival across gastrointestinal and other malignancies^1–4^. Their benefit reflects the integration of complementary specialty perspectives into a shared decision. Yet their resource intensity limits global accessibility, particularly in settings with specialist shortages, and creates delays that may affect treatment timeliness^5–8^. Large language models (LLMs) have attracted interest as potential decision-support adjuncts: across multiple tumour types, agreement rates of 50–87% have been reported between LLM recommendations and actual MDT decisions^9–12^, with 77–85% concordance for GPT-4-class models specifically in gastrointestinal oncology^13,14^. Systematic reviews confirm, however, that performance is sensitive to tumour type, prompt design, and evaluation methodology, and that accuracy gains across model generations are often smaller than anticipated^15^.

Most published LLM evaluations treat the model as a monolithic system, applying a single undifferentiated prompt and measuring output against the board’s collective decision. This approach cannot reveal whether the model is capable of specialty-specific reasoning or whether it retrieves plausible oncological text regardless of clinical framing. Role-based prompting—instructing the model to adopt the perspective of a surgical, medical, or radiation oncologist—offers a mechanism to probe this distinction. While role prompting can improve performance on reasoning tasks when thematically aligned with the problem^16^, large-scale evaluation across 162 roles found no consistent accuracy gains and occasional performance decrements^17^. Whether role assignment induces genuinely distinct clinical decision-making in oncology, or merely produces specialty-appropriate language around an otherwise invariant recommendation, has not been systematically tested.

Moreover, ensemble aggregation of role-specific outputs—using majority voting to combine recommendations from multiple specialist personas—has been widely applied in clinical machine learning and consistently outperforms individual models in oncology decision support^18–23^.

We addressed these questions by applying five zero-shot prompting frameworks to GPT-5 across 100 gastrointestinal oncology cases adjudicated by a real MDT, with a majority-vote ensemble as a sixth aggregated comparator.

This study was designed to answer the following research questions:

- Does specialty-specific role prompting induce genuinely distinct clinical output structures in a state-of-the-art LLM, or does it produce role-appropriate language around an otherwise invariant recommendation?
- Does explicit multi-expert deliberation within a single structured prompt improve concordance and output diversity relative to both individual specialist persona prompting and standard MDT simulation?
- Does ensemble aggregation of role-specific outputs via majority voting improve concordance with MDT recommendations beyond individual framework performance?

The main contributions of this work are: (1) the first systematic, multi-dimensional characterisation of role prompting effects in gastrointestinal oncology LLM evaluation, spanning concordance, linguistic analysis, embedding geometry, and entropy; (2) an empirical comparison of six distinct prompting frameworks—including specialist personas, structured deliberation, and majority-vote ensemble—against real MDT decisions; and (3) an empirical foundation for understanding when and why architectural role separation may be necessary to achieve genuine output diversity, with direct implications for the design of multi-agent clinical AI systems.

## Results

### Study cohort

One hundred gastrointestinal cancer cases were analysed across five tumour types (20 cases each). Surgery (29%) and systemic therapy (29%) were the most frequently recommended treatment modalities overall (Supplementary Table 1). Tumour-specific distributions varied substantially: pancreatic cancer was predominantly managed surgically (55%), oesophageal cancer primarily received multimodal approaches (35%), and hepatobiliary cancer was split between surgery and systemic therapy (35% each).

### Overall concordance rates and framework comparison

**Fig. 1:**
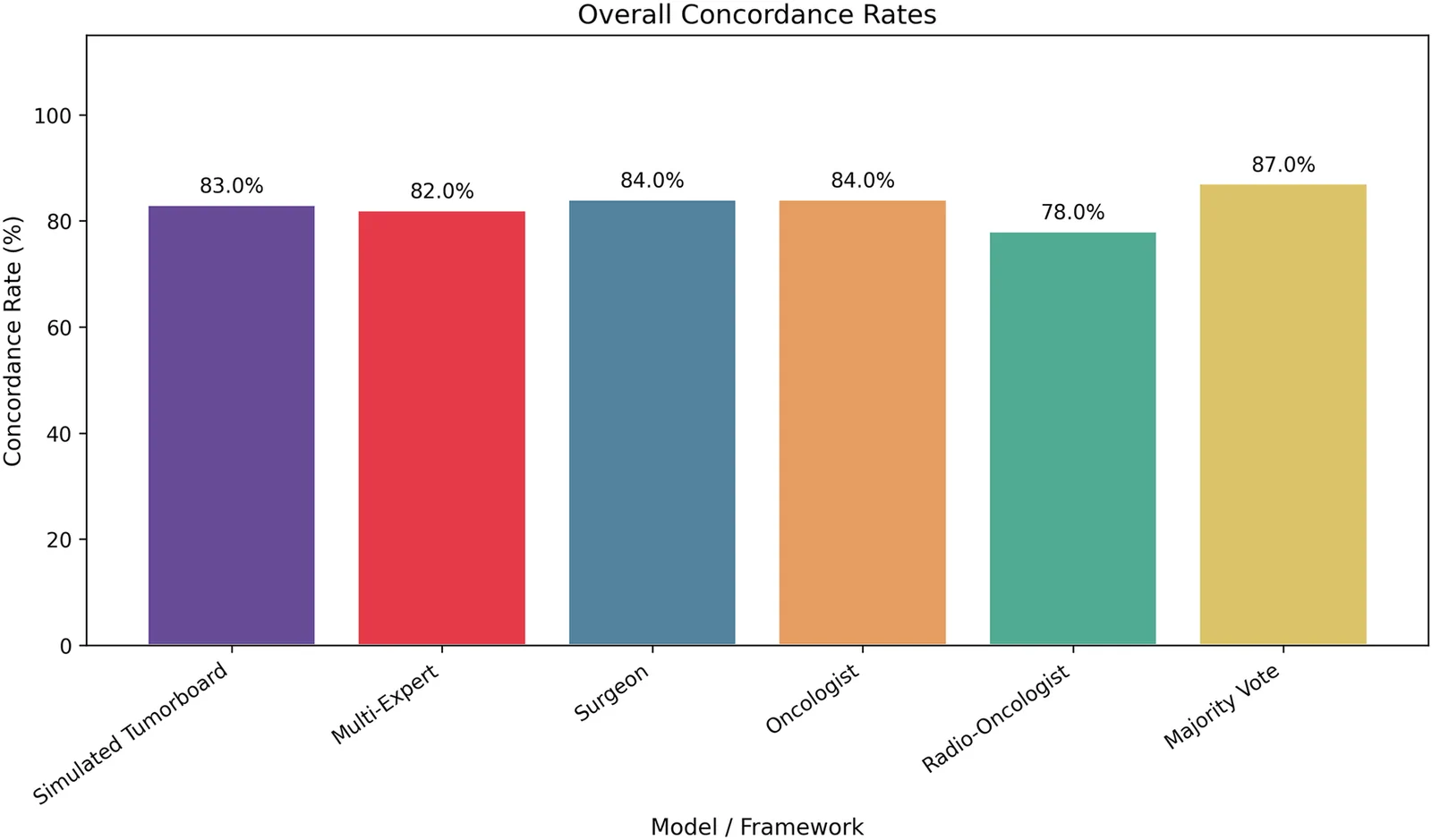
Concordance rates of GPT-5 frameworks with tumour board recommendations. Bars show overall percentage concordance for individual specialist roles, multi-expert deliberation, and majority-vote ensemble. The majority-vote ensemble achieved the highest concordance (87%), with individual roles ranging from 78–84%. Statistical comparisons are reported in the text.

Concordance rates with MDT recommendations ranged from 78% to 87% across the six frameworks (Table 1 and Fig. 1). The majority vote ensemble achieved the highest concordance at 87% (Framework 6: 87/100; 95% CI: 79.0%–92.2%), representing a 4-percentage-point improvement over the simulated multidisciplinary tumour board (Framework 1: 83%, 83/100; 95% CI: 74.5%–89.1%).

**Table 1:** Overall concordance rates with MDT recommendations

Framework	*N*	Correct	Concordance (%)	95% CI lower	95% CI upper
Simulated Tumour Board	100	83	83.0	74.5%	89.1%
Multi-expert Deliberation	100	82	82.0	73.3%	88.3%
Surgical Oncologist	100	84	84.0	75.6%	89.9%
Medical Oncologist	100	84	84.0	75.6%	89.9%
Radiation Oncologist	100	78	78.0	68.9%	85.0%
Majority Vote Ensemble	100	87	87.0	79.0%	92.2%

Among individual specialist role frameworks, the surgical and medical oncologist achieved identical concordance rates of 84% (84/100; 95% CI: 75.6%–89.9%), followed by the multi-expert deliberation framework (82%, 82/100; 95% CI: 73.3%–88.3%) and the radiation oncologist (78%, 78/100; 95% CI: 68.9%–85.0%). Confidence intervals showed considerable overlap across frameworks.

Cochran’s Q test did not reveal statistically significant differences across the six frameworks (Q = 8.462, df = 5, *p* = 0.133). Among pairwise McNemar comparisons, only the contrast between radiation oncologist and majority vote reached nominal statistical significance (χ² = 7.11, *p* = 0.008); after Bonferroni correction for 15 simultaneous comparisons (corrected α = 0.0033), no pairwise contrast survived (p_corr = 0.115) (Supplementary Table 2).

Post-hoc power analysis revealed low power across most framework contrasts (0.05–0.41 for 14 of 15 comparisons; Supplementary Fig. 1 and Supplementary Table 3), with one exception discussed below. For the clinically relevant comparison between majority vote and simulated MDT, post-hoc power was 0.24, with only 10 discordant cases (7 favouring majority vote, 3 favouring simulated MDT). The only comparison achieving adequate power was majority vote versus radiation oncologist (power = 0.85), where all 9 discordant cases exclusively favoured the ensemble. These findings indicate that the non-significant Cochran’s Q result reflects genuine convergence in framework performance rather than systematic underpowering.

Pairwise inter-framework agreement, quantified using Cohen’s kappa across all framework pairs including the MDT reference standard, is reported in Supplementary Note 5.

### Performance by tumour type and consultation context

Concordance varied markedly by tumour type: oesophageal cancer achieved 95–100% concordance across all frameworks, whereas colorectal malignancies showed the widest performance range (65–80%). Concordance rates were comparable between initial presentations and follow-up consultations (76–86% vs 80–98%, respectively). Full tumour-type and consultation-type stratified results, including per-framework Wilson confidence intervals and confusion matrices, are reported in Supplementary Sections S6 and S7.

### Role authenticity analysis

**Fig. 2:**
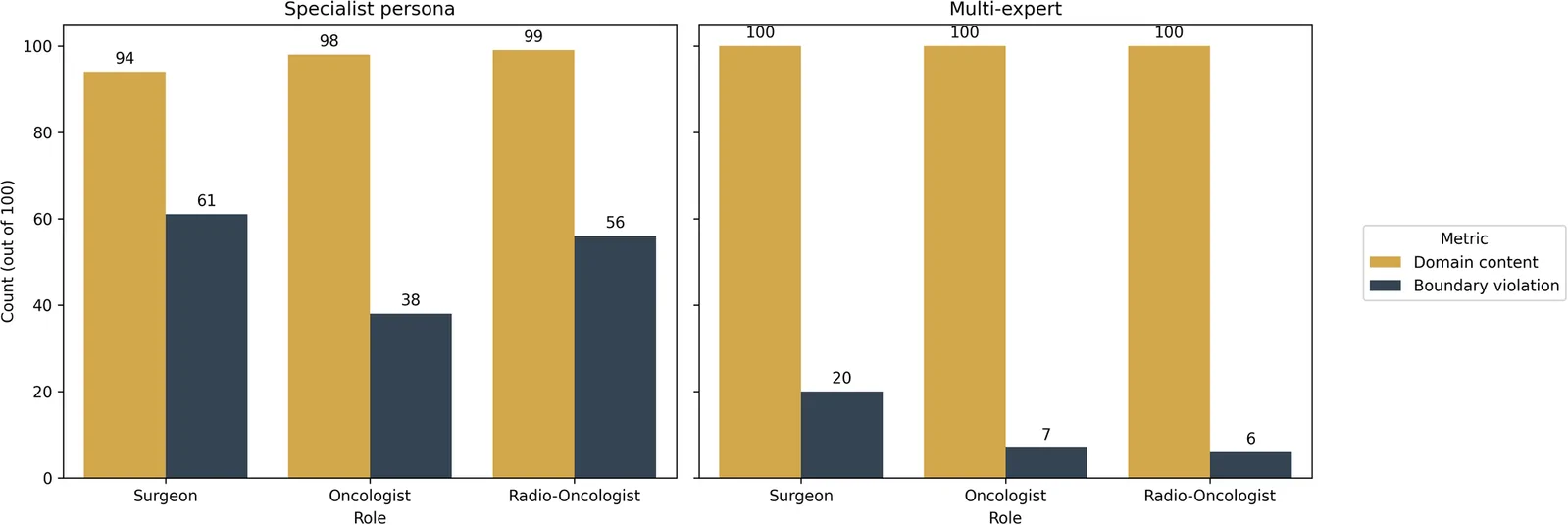
Domain-specific content generation and role boundary adherence across prompting frameworks. Comparison of specialist persona prompting (Frameworks 3–5) and multi-expert deliberation prompting (Framework 2) for three simulated oncology specialists: surgical oncologist, medical oncologist, and radiation oncologist. Bars represent the proportion of responses containing specialty-specific domain terminology (domain content) and cross-disciplinary treatment decisions outside the assigned specialty (boundary violations).

GPT-5 consistently incorporated specialty-characteristic terminology under role-specific prompting (Fig. 2): surgical oncologist outputs contained surgery-specific content in 94% of cases, medical oncologist outputs in 98%, and radiation oncologist outputs in 99%. Despite this high level of domain-aligned language, boundary adherence was substantially more variable. Cross-disciplinary treatment recommendations were observed in 61% of surgical oncologist outputs, 56% of radiation oncologist outputs, and 38% of medical oncologist outputs. Boundary violation classification followed pre-specified operational definitions; all cases were unambiguously classifiable and no inter-rater discordance was observed during annotation.

Under multi-expert deliberation, domain-specific terminology became universal across all simulated specialists (100% for all roles), while boundary violations decreased markedly—to 20% for the surgical oncologist, 7% for the medical oncologist, and 6% for the radiation oncologist. This represents a relative reduction of 67–84% in cross-disciplinary decision leakage compared to single-specialty prompting. Thus, although both paradigms produced highly domain-specific language, explicit role separation substantially constrained cross-disciplinary treatment recommendations.

These findings suggest that under single-specialty prompting, the model integrates knowledge from multiple clinical domains despite adopting role-specific language. Structured multi-expert deliberation, by contrast, produces clearer role compartmentalisation, constraining responses more effectively within professional scope.

Point-biserial correlation analysis restricted to specialist persona frameworks (Frameworks 3–5) revealed no significant association between specialty-specific content presence and recommendation concordance for any role: surgical oncologist (*r* = 0.005, *p* = 0.964), medical oncologist (*r* = −0.062, *p* = 0.538), and radiation oncologist (*r* = −0.053, *p* = 0.598). Cross-disciplinary content showed no consistent relationship with concordance, with the exception of a weak positive association in the radiation oncologist framework (*r* = 0.259, *p* = 0.009); after Bonferroni correction for six simultaneous comparisons (corrected α = 0.0083), this association was no longer statistically significant (p_corr = 0.056), and the small effect size precludes clinical interpretation.

Under specialist persona prompting (*n* = 300 independent responses, one per role per case), simultaneous presence of both role-specific and cross-disciplinary content was the most frequent pattern (148/300, 49.3%), followed by role-specific content only (143/300, 47.7%) (Table 2 and Supplementary Table 5). Concordance was consistently higher for responses containing both content types (87.8%) compared to role-specific content only (75.5%), a pattern observed across all three roles (surgeon: 85.7% vs 81.6%; oncologist: 91.9% vs 78.7%; radiation oncologist: 87.3% vs 65.9%).

**Table 2:** Concordance with MDT treatment recommendations stratified by content category and prompting framework

Category	Concordance with MDT — Specialist Persona, n/N (%)				Concordance with MDT — Multi-Expert Deliberation, n/N (%)			
	Surgeon (*n* = 100)	Oncologist (*n* = 100)	Radiation Oncologist (*n* = 100)	Overall (*n* = 300)	Surgeon (*n* = 100)	Oncologist (*n* = 100)	Radiation Oncologist (*n* = 100)	Overall (*n* = 300)
Role-specific only	31/38 (81.6%)	48/61 (78.7%)	29/44 (65.9%)	108/143 (75.5%)	64/80 (80.0%)	75/93 (80.6%)	76/94 (80.9%)	215/267 (80.5%)
Cross-disciplinary only^a^	4/5 (80.0%)	1/1 (100.0%)	1/1 (100.0%)	6/7 (85.7%)	—	—	—	—
Both role-specific and cross-disciplinary	48/56 (85.7%)	34/37 (91.9%)	48/55 (87.3%)	130/148 (87.8%)	18/20 (90.0%)	7/7 (100.0%)	6/6 (100.0%)	31/33 (93.9%)
Neither^a^	1/1 (100.0%)	1/1 (100.0%)	—	2/2 (100.0%)	—	—	—	—

Values indicate the number of concordant recommendations over total responses within each category (*n*/*N*) and the corresponding percentage. Cells with *n* < 5 should be interpreted cautiously. Specialist persona prompting (Frameworks 3–5): *n* = 300 independent responses, one per role per case, concordance evaluated at the individual response level. Multi-expert deliberation (Framework 2): *n* = 300 individual reasoning paths across 100 cases (three paths per case); concordance reflects the final consensus recommendation generated from those paths, with content classification applied to the individual reasoning paths.

^a^Categories with *n* < 5 (cross-disciplinary content only and neither) are reported for completeness but should be interpreted with caution given distributional instability at small sample sizes.

Under multi-expert deliberation, content classification was applied to the individual role-specific reasoning paths within each structured output (*n* = 300 paths across 100 cases, three per case); the concordance outcome reflects the consensus recommendation generated from those paths. Role-specific content only became the dominant pattern (267/300, 89.0%), with cross-disciplinary content appearing in at least one reasoning path in only 33 cases (11.0%). Despite this, consensus concordance remained higher for cases where at least one reasoning path contained cross-disciplinary content (93.9% vs 80.5%), suggesting that boundary expansion within individual role paths may facilitate integrative reasoning that benefits the final consensus — consistent with the pattern observed under single-specialty prompting.

**Fig. 3:**
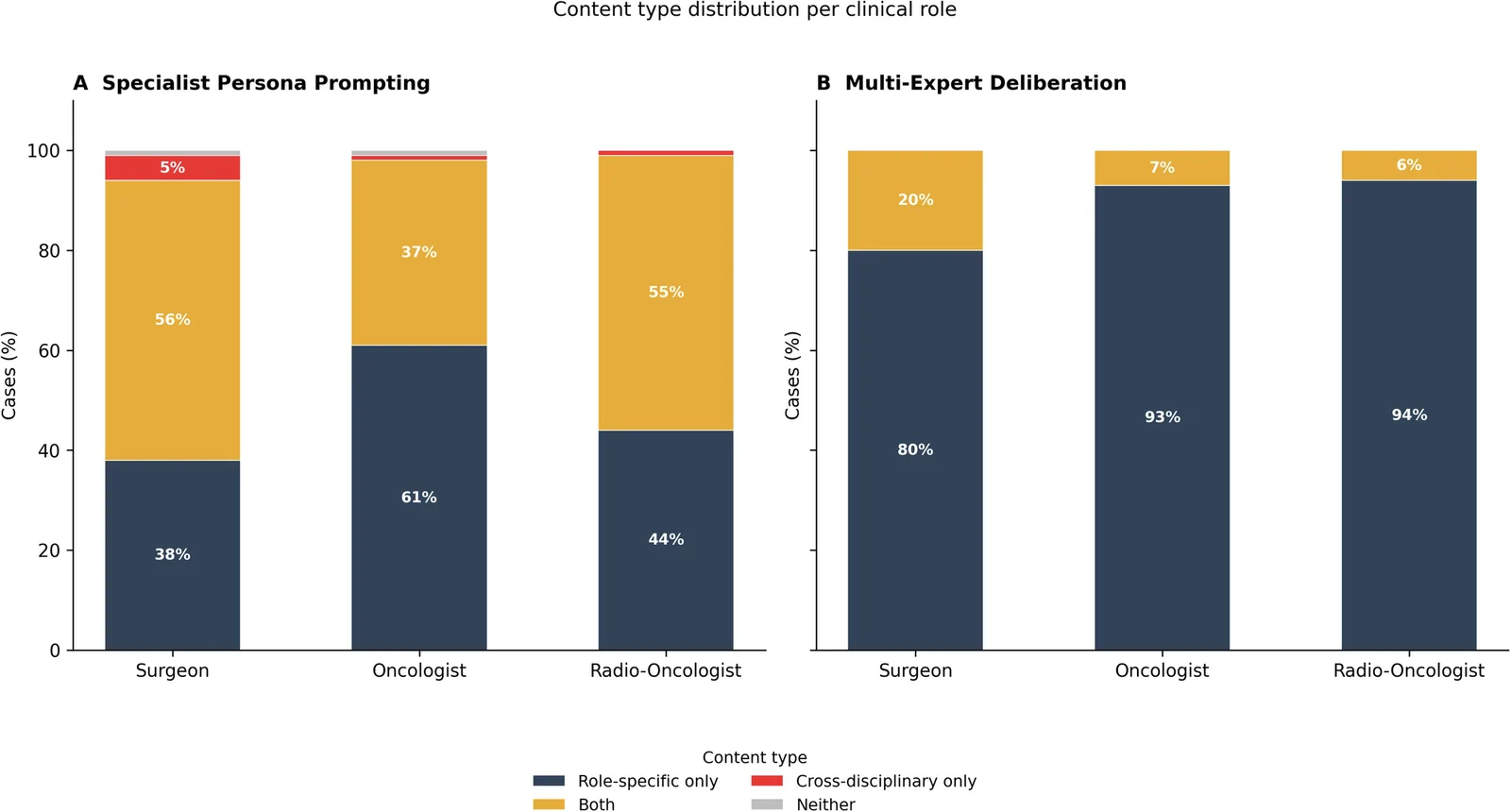
Content type distribution per clinical role under specialist persona prompting and multi-expert deliberation. Stacked bars show the proportion of cases per role classified into four mutually exclusive content categories: role-specific content only (dark navy), both role-specific and cross-disciplinary content (amber), cross-disciplinary content only (red), and neither (grey). **A** (specialist persona prompting) illustrates a heterogeneous mix of content types across all three roles, with combined role-specific and cross-disciplinary content representing the most frequent pattern. **B** (multi-expert deliberation) shows a marked shift towards role-specific content dominance, reflecting the stronger role compartmentalisation imposed by explicit structural separation. Percentage labels are shown for segments ≥ 5%. *n* = 100 cases per role per condition.

Taken together, these findings suggest that cross-disciplinary content, rather than being purely a marker of role boundary violation, may carry complementary clinical information that supports recommendation accuracy—under both single-specialty prompting and, at the level of individual reasoning paths, within multi-expert deliberation (Fig. 3).

Shannon entropy analysis confirmed low inter-specialist decision variability overall (mean: 0.277 bits; median: 0 bits), with complete unanimity across all three specialist roles in 72 cases (72%). In only 2 of 100 cases did majority voting produce an incorrect recommendation while at least one individual specialist role was correct. Entropy varied systematically by tumour type: hepatobiliary tumours exhibited the highest inter-specialist uncertainty (mean: 0.526 bits), followed by colorectal (0.401 bits), gastric (0.275 bits), and pancreatic and oesophageal cancers (0.092 bits each).

### Semantic embedding analysis

**Fig. 4:**
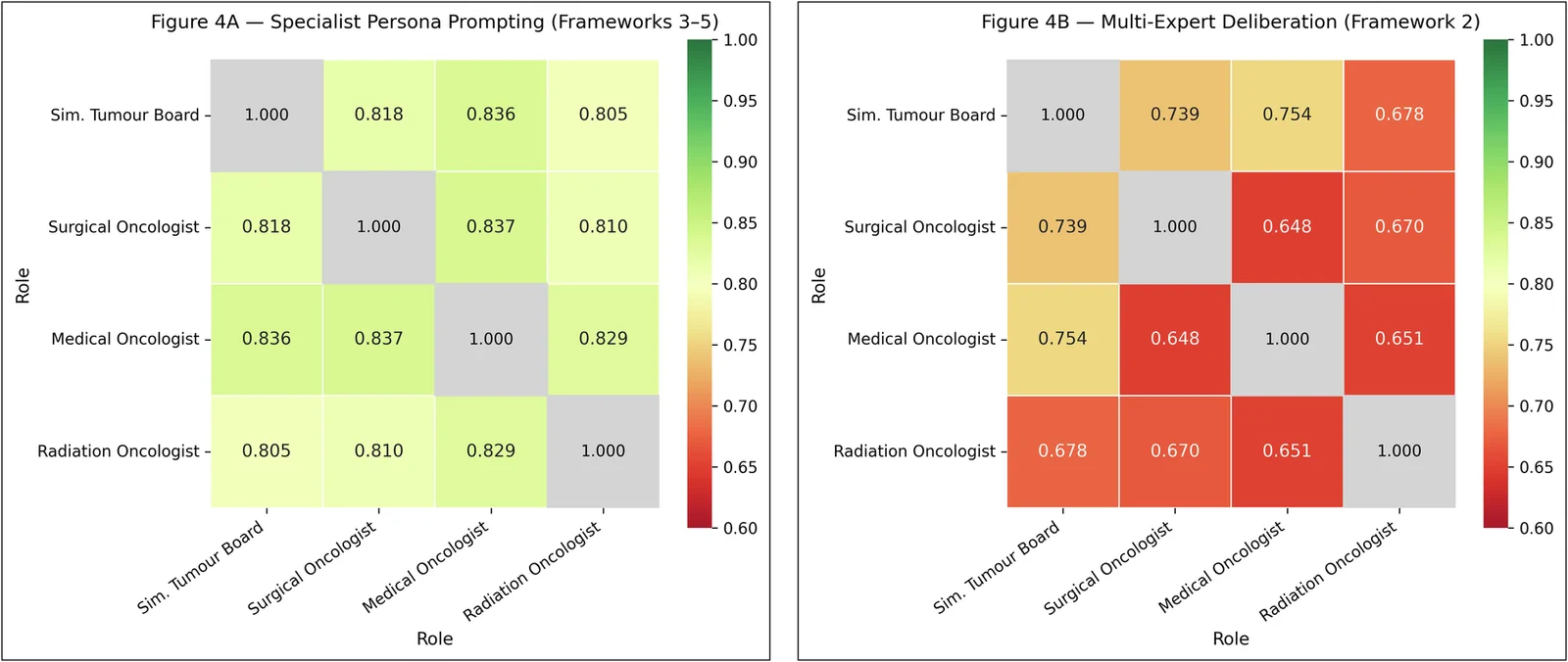
Pairwise cosine similarity of GPT-5 role-specific embeddings under two prompting frameworks. Heatmaps show mean pairwise cosine similarity between role-specific embeddings under specialist persona prompting (**A**, Frameworks 3–5) and multi-expert deliberation (**B**, Framework 2). Each cell displays the mean cosine similarity between the two corresponding role embedding distributions (annotated numerically), colour-coded from low (red/yellow) to high (green) similarity. Diagonal cells represent self-similarity (1.000). Under specialist persona prompting, inter-role similarity was uniformly high (range: 0.805–0.836), reflecting substantial semantic overlap across role conditions. Multi-expert deliberation markedly reduced inter-role similarity (range: 0.649–0.755), indicating stronger role compartmentalisation under structured deliberative prompting.

In the specialist persona condition, mean pairwise cosine similarities ranged from 0.805 to 0.836, indicating high global semantic coherence across specialist personas (Fig. 4A). Multi-expert deliberation (Framework 2) markedly reduced inter-role similarity (range: 0.651–0.755) (Fig. 4B).

**Fig. 5:**
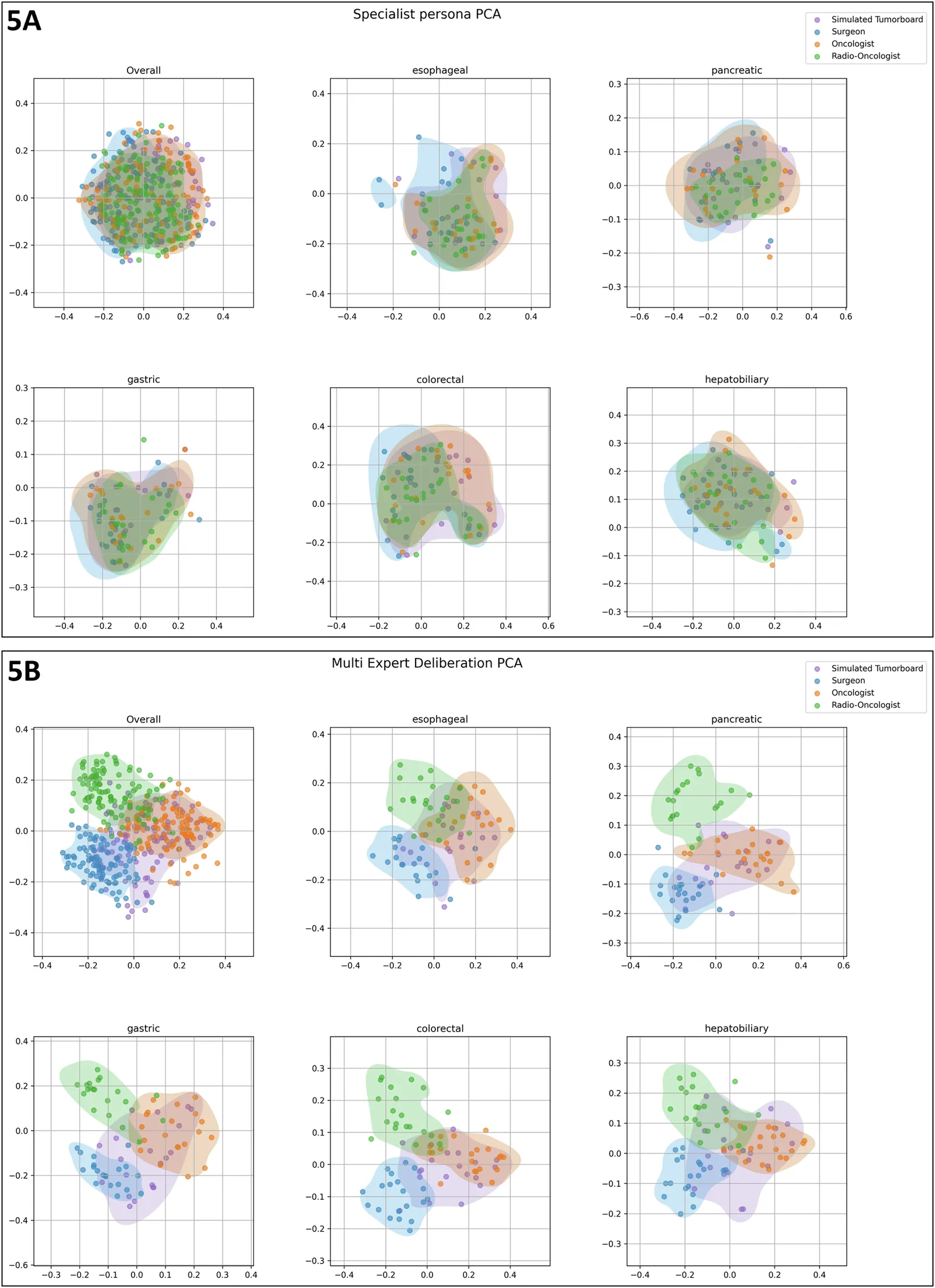
Principal component analysis (PCA) of role-specific GPT-5 embeddings under two prompting paradigms. Scatter plots show the first two principal components of high-dimensional embeddings, with colours indicating specialist role: Simulated Tumour Board (purple), Surgeon (blue), Oncologist (orange), Radiation Oncologist (green). Kernel density estimates (shaded contours) highlight regions of higher embedding density. The left-most panel in each row displays PCA across all cases; remaining panels show tumour-type-specific projections (oesophageal, pancreatic, gastric, colorectal, hepatobiliary). **A** Specialist Persona Prompting (Frameworks 3–5). Embeddings generated under single-specialty role prompting (PC1: 6.4%, PC2: 5.1% of total variance). Substantial overlap across role centroids reflects limited role-attributable variance (Kruskal–Wallis H = 19.79, *p* < 0.001, η² = 0.049). **B** Multi-Expert Deliberation (Framework 2). Embeddings generated under structured multi-expert deliberation prompting, using the same layout as **A**). Role separation increased markedly (PC1: H = 222.26, *p* < 0.001, η² = 0.554; PC2: H = 241.29, *p* < 0.001, η² = 0.581), with more than half of embedding variance attributable to persona assignment. Centroid distances ranged from 0.286 to 0.326, compared to 0.192–0.241 under specialist persona prompting.

For specialist persona embeddings, PCA explained 6.4% (PC1) and 5.1% (PC2) of total variance (Fig. 5A). Kruskal–Wallis testing confirmed statistically significant role separation on PC1 (H = 19.79, *p* < 0.001), but effect size was small (η² = 0.049), indicating limited role-attributable variance. Under multi-expert deliberation (Fig. 5B), separation increased markedly (PC1: H = 222.26, *p* < 0.001, η² = 0.554; PC2: H = 241.29, *p* < 0.001, η² = 0.581), with more than half of embedding variance attributable to persona assignment. Full tumour-type-stratified PCA results and centroid separation matrices are provided in Supplementary Note 9.

In the full embedding space, centroid distances in the specialist persona condition ranged from 0.192 (oncologist vs simulated tumour board) to 0.241 (simulated tumour board vs radiation oncologist). Under multi-expert deliberation, separation increased to 0.286–0.326. Jensen–Shannon divergence between role distributions ranged from 0.018 to 0.097 in both conditions, with role pairings of maximal divergence shifting between prompting paradigms. Full embedding geometry results are reported in Supplementary Note 10.

Case-level cosine similarity between specialist persona and multi-expert deliberation embeddings indicated moderate drift (surgeon: 0.797 ± 0.061; simulated tumour board: 0.792 ± 0.054; oncologist: 0.758 ± 0.070; radiation oncologist: 0.750 ± 0.063). At the centroid level, drift was minimal (cosine similarity 0.958–0.980), indicating that aggregate role representations remained stable despite case-level variability. Full drift statistics are reported in Supplementary Note 10.

Composite persona stability metrics are reported in full in Supplementary Note 11. Briefly, the PSI was highest for the medical oncologist (0.805), reflecting the most balanced combination of semantic identity preservation, role specificity, and boundary control, followed by the surgical and radiation oncologist (both 0.763). The CRI followed the same ordering (medical oncologist: 0.810; surgical oncologist: 0.768; radiation oncologist: 0.765). Bootstrap confidence intervals overlapped across all roles, confirming that no persona demonstrated statistically superior stability. The Clinical Risk Penalty Score was lowest for the medical oncologist (0.236) and highest for the surgical oncologist (0.390), consistent with the latter’s higher boundary violation rate (61%). PSI rank ordering was invariant across all weight perturbations (±10–20%), and PSI and CRI showed strong convergent validity (Spearman ρ = 0.94), supporting the robustness of these descriptive summaries. Component contribution analysis indicated that boundary control and accuracy collectively accounted for 93% of PSI variance, with specificity rate contributing 7%; entropy stability contributed negligibly to CRI variance (0.02%), indicating that this component adds minimal discriminative information in the current dataset.

## Discussion

GPT-5 reliably adopted specialty-characteristic language across surgical, medical, and radiation oncology personas, yet this surface adaptation did not translate into meaningfully distinct clinical decision structures. Concordance with MDT recommendations remained within a narrow range (78–87%) across all six frameworks, and no inter-framework differences survived correction for multiple comparisons—findings consistent with prior LLM oncology evaluations^9–14,24^. Throughout, concordance denotes category-level agreement with the documented MDT consensus—the established reference standard for complex oncological care^1–4^—against which all frameworks are compared. These results reflect behavioural and representational signatures of role prompting rather than its internal reasoning mechanisms, and should be interpreted accordingly.

Specialty-characteristic terminology appeared in nearly all outputs (97–100%), but its presence was not correlated with recommendation accuracy (*p* > 0.05 across roles). This dissociation is consistent with established findings: although persona prompting can improve zero-shot reasoning when thematically aligned with the task^16^, systematic testing across 162 roles found no consistent accuracy gains and occasional decrements^17^^,25^, a pattern reproduced in clinical benchmarks^26^.

Embedding analysis reinforced this interpretation. Under specialist persona prompting, pairwise cosine similarities were high (0.805–0.836) and centroid separation moderate (0.192–0.241), suggesting proximity within a shared semantic space rather than clearly distinct specialty clusters. PCA explained 6.4% (PC1) and 5.1% (PC2) of total variance—values that reflect the distributional properties of high-dimensional embedding spaces rather than poor dimensionality reduction, and whose interpretive significance is best assessed relative to other prompting conditions rather than in isolation. Role separation on PC1 reached statistical significance (H = 19.79, *p* < 0.001), but effect size was small (η² = 0.049), confirming that role-attributable variance was limited under this framework. Multi-expert deliberation produced a markedly different geometric signature: inter-role cosine similarity fell to 0.651–0.755, centroid separation increased to 0.286–0.326, and persona-attributable variance exceeded 50% along principal components (PC1: η² = 0.554; PC2: η² = 0.581)—a more than 10-fold increase relative to specialist persona prompting. This contrast demonstrates that explicit role separation induces substantially greater geometric separation in output embedding space—a representational shift not achieved by persona prompting alone, though its relationship to underlying reasoning processes remains indirect. Given that embedding distance reflects statistical co-occurrence rather than causal cognitive processes^27,28^, however, greater geometric separation should not be conflated with deeper clinical reasoning differentiation. Mechanistically, the greater centroid separation and higher persona-attributable variance observed under multi-expert deliberation reflect shifts in the distributional statistics of the generated text—lexical, syntactic, and topical co-occurrence patterns captured by the embedding model—rather than differentiation in the underlying inferential process. The dissociation is made explicit by the accuracy data: this pronounced representational restructuring produced no corresponding gain in MDT concordance, confirming that output-space geometry and clinical decision quality are dissociable properties.

Taken together, persona prompting appears to modulate expression style rather than restructure the model’s underlying statistical decision patterns — consistent with characterisations of LLM clinical reasoning as distribution-constrained pattern recognition rather than flexible abstract inference^26,29^. Multi-expert deliberation produces representationally distinct outputs, but as the accuracy and boundary analyses below demonstrate, geometric separation and clinical superiority are dissociable.

Composite robustness metrics ranged from 0.763 to 0.810 across roles, with overlapping bootstrap confidence intervals. No specialist persona demonstrated superior decision stability—a finding aligned with prior work showing that large language models reproduce shared statistical knowledge representations rather than independent reasoning architectures^26,29^.

Entropy analysis reinforced this pattern. Mean inter-specialist Shannon entropy was 0.277 bits (median = 0 bits), indicating that more than half of cases produced identical recommendations regardless of persona assignment. This degree of convergence is consistent with pattern-based clinical AI inference, where predictions emerge from learned statistical associations rather than dynamically differentiated reasoning strategies^26,28^, and resembles established clinical decision support paradigms where reliability derives from stable pattern matching rather than mechanistic simulation of specialist cognition^28,30^.

Rank ordering of composite stability metrics was invariant under weight perturbation across all tested levels, and strong PSI–CRI convergence (Spearman ρ = 0.94) supports the structural consistency of both measures. Their convergent values across roles, alongside the embedding and entropy evidence, collectively support the interpretation that persona assignment modulates surface behaviour rather than underlying decision architecture.

Cross-disciplinary treatment recommendations outside the assigned specialty appeared in 38–61% of single-specialist outputs—frequent boundary expansion despite specialty-specific language. Paradoxically, responses containing both specialty-specific and cross-disciplinary content achieved the highest concordance (85.7–91.9%), exceeding role-specific content alone. In this configuration, the model does not behave as an isolated clinician but rather as a compressed tumour board operating within a single persona: when a “surgeon” also proposes systemic or radiotherapy considerations, the response implicitly integrates multiple domain priors.

It is worth noting that boundary violations were defined as autonomous treatment decisions belonging to another specialty—not as contextual acknowledgement of multidisciplinary findings—measuring professional scope realism rather than interdisciplinary awareness.

The contrast with multi-expert deliberation is instructive. Enforcing explicit role separation markedly reduced boundary violations (surgeon 61% → 20%; medical oncologist 38% → 7%; radiation oncologist 56% → 6%), while domain-specific terminology remained universal. Embedding analysis revealed correspondingly greater representational separation, with persona-attributable variance exceeding 50% along principal components—suggesting that explicit role separation induces substantially greater geometric divergence in output space.

Still, this structural specialisation did not improve recommendation accuracy. Greater geometric separation therefore reflects representational compartmentalisation rather than enhanced clinical inference. Enforcing strict persona boundaries produces outputs more closely resembling real-world professional roles, but may reduce the implicit multidisciplinary integration that supports higher concordance under single-persona prompting. Consistent with the clinical AI literature, apparent specialisation in medical AI systems often reflects overlapping domain knowledge distributions rather than distinct inferential mechanisms^30^, and emerging evidence suggests that optimal performance may require flexible cross-domain integration rather than rigid role compartmentalisation^27,31,32^.

Majority voting achieved the highest concordance (87%; 95% CI 79.0–92.2%), representing a 4-percentage-point improvement over the simulated tumour board (83%). Although not statistically significant, this corresponds to an observed absolute reduction in discordant cases (17 to 13), consistent with classical bias–variance tradeoff principles, where aggregation improves predictive stability by reducing variance-driven error while preserving systematic decision structure^33^. Similar behaviour has been reported in oncology AI decision support systems, where ensemble aggregation improves reliability despite limited model diversity^22,34^.

Critically, gains occurred despite high semantic and decision-level correlation across roles (κ = 0.612–0.720 against MDT; κ = 0.724–0.898 inter-role), indicating that improvement was driven by partial error decorrelation rather than emergent output diversity—consistent with ensemble learning theory and prior oncology AI evidence^18–21,23,25,33,35^.

The finding that role prompting modulates linguistic style but not reasoning structure carries a specific architectural implication: the locus of specialty differentiation matters. Under single-prompt persona assignment, GPT-5 operates as an inherently multidisciplinary system—drawing on shared statistical representations regardless of the role label applied. The contrast with multi-expert deliberation is instructive: explicit structural separation of reasoning paths produced a more than tenfold increase in role-attributable embedding variance (η² from 0.049 to 0.554), suggesting that substantially greater output-space divergence requires architectural enforcement, not merely prompt-level instruction—though whether this reflects deeper reasoning differentiation or representational restructuring at the output level cannot be determined from embedding geometry alone.

This distinction is directly relevant to the rapidly developing field of multi-agent clinical AI. Recent frameworks that deploy separate, independent agents per specialty—each with dedicated context, knowledge retrieval, and conflict resolution mechanisms—have demonstrated performance gains over single-agent baselines in oncology decision support. A multi-agent conversation framework inspired by MDT discussions outperformed single LLM models in both primary and follow-up rare disease consultations, with optimal performance achieved using four specialist agents and a supervisor agent^36^. EvoMDT, a self-evolving multi-agent system published in npj Digital Medicine, employs domain-specific agents with structured knowledge retrieval and a consensus protocol for conflict resolution, with evaluation spanning six oncology benchmarks and four real-world datasets^37^.

Our results provide an empirical basis for understanding why such architectures outperform persona-prompted single models: the performance advantage is unlikely to derive from role labels alone, but from the combination of separated contexts, independent inference, and explicit disagreement mechanisms that force genuine perspective divergence. Our embedding findings suggest that even this approach produces greater representational separation than single-prompt persona prompting, but that further gains will likely require decentralised context and explicit conflict resolution beyond role assignment.

Specialty-specific language should therefore not be interpreted as evidence of specialty-specific reasoning. Future performance gains will likely require architectural diversification—including retrieval-augmented generation, domain-specific fine-tuning, and agentic multi-specialist workflows with independent context and structured consensus mechanisms—rather than advanced persona engineering alone^37–40^.

Several limitations should be acknowledged. First, all frameworks derive from a single model (GPT-5): observed role diversity represents prompt-induced variation within one parameter distribution rather than true ensemble diversity across independently trained models, limiting extrapolation to multi-model architectures. Second, statistical power to detect pairwise differences was constrained by the small number of discordant cases per comparison (3–15 pairs); the 4-percentage-point ensemble advantage should be interpreted as an effect size estimate rather than a confirmed superiority finding. Third, MDT concordance measures alignment with expert consensus rather than absolute clinical correctness, as multiple valid treatment pathways may exist for any given case. Fourth, vignettes were constructed in German to match institutional documentation; performance may differ from English-language settings, representing a confound not directly assessed. Fifth, the training knowledge cutoff of GPT-5 (September 2024) postdates the clinical vignettes used in this study (March 2022–December 2023): direct case-level data leakage is implausible given that vignettes were never publicly disclosed, but the model was necessarily trained on the same clinical guideline landscape that informed MDT decisions—a shared knowledge base that is a prerequisite for meaningful concordance evaluation rather than a methodological confound, yet precludes attributing agreement to independent reasoning rather than guideline retrieval. Sixth, embedding and entropy metrics capture statistical structure rather than cognitive reasoning, and manual annotation of role authenticity introduces potential classification bias. Seventh, surgical decision-making showed the greatest framework sensitivity (59–76% concordance), likely reflecting the multi-factorial nature of resectability evaluation—integrating imaging interpretation, patient fitness, and surgical judgement—that vignette-based assessment incompletely captures; concordance figures for this domain should therefore be interpreted with particular caution.

GPT-5 demonstrates strong linguistic adaptation to clinical personas but limited specialisation in output structure, behaving as an inherently multidisciplinary decision system drawing on shared underlying representations rather than specialty-segmented inference. Ensemble aggregation produced an observed absolute reduction in discordant cases that did not reach statistical significance. These findings support LLMs as clinical decision support tools rather than autonomous specialist simulators, and suggest that future progress will depend on architectural solutions — including multi-agent systems with separated contexts and explicit conflict resolution — rather than on increasingly sophisticated role prompting strategies. Future work should assess whether the observed dissociation between linguistic adaptation and output diversity generalises across model families — including smaller and open-source LLMs — and across languages beyond German, to establish the external validity of these findings.

## Methods

### Study design and patient selection

This retrospective study was approved by the local Ethics Committee (approval number 273/2024BO1) and conducted in accordance with the Declaration of Helsinki. The requirement for informed consent was waived by the Ethics Committee due to the retrospective and anonymised nature of the study. Gastrointestinal cancer cases presented at the institutional MDT between March 2022 and December 2023 were screened for eligibility. Cases were excluded if patients were enrolled in clinical trials, primarily referred for trial participation, or presented with mixed diagnoses. Five tumour groups were defined: oesophageal, gastric, pancreatic, hepatobiliary, and colorectal cancer. For each group, 20 cases were selected by simple random sampling (10 initial presentations and 10 follow-up consultations), with ineligible cases replaced as necessary to achieve balanced representation.

### Data collection and anonymization

Clinical information was extracted from standardised MDT documentation templates, including patient demographics, ECOG performance status, primary diagnosis with histological confirmation, relevant comorbidities, prior medical history, current concomitant conditions, and results of all completed diagnostic workup available at the time of MDT presentation (including endoscopy, cross-sectional imaging, and functional imaging where clinically indicated). The specific clinical question posed to the board and the official recommendation were also recorded. Vignette content was not modified or simplified prior to LLM input; inputs thus reflected the complete information available to the tumour board at the time of presentation. All data were fully anonymised through removal of personal identifiers and standardisation of temporal information. The resulting anonymised clinical vignettes served as the standardised input for all prompting conditions.

### Large language model framework

We applied GPT-5 (OpenAI; model version gpt-5-2025-08-07; training knowledge cutoff: September 2024) across 100 gastrointestinal oncology cases using five distinct zero-shot prompting frameworks and a majority-vote ensemble; all prompts required responses in German to match the language of institutional MDT documentation. Exact prompt templates are provided in the Supplementary Material Section S1.

Prompt design followed established principles for zero-shot clinical prompting: a system prompt defined the role and epistemic frame, while the user prompt constrained output format, language, and decision scope. Critically, all frameworks explicitly prohibited deferral to further multidisciplinary discussion, ensuring that each framework produced an actionable treatment recommendation directly comparable to the MDT reference standard. The multi-expert deliberation framework (Framework 2) was inspired by the self-consistency reasoning paradigm, in which multiple independent reasoning paths are sampled before synthesis into a final answer^41^. No few-shot examples were provided; consistent with prior work in comparable clinical settings reporting no consistent benefit of few-shot over zero-shot prompting^42,43^ and no improvement in accuracy with few-shot learning in clinical guideline interpretation^44^, zero-shot prompting was considered the more appropriate and reproducible baseline.

Framework 1: Standard MDT Simulation. The model was instructed to act as a complete multidisciplinary tumour board including surgical oncology, medical oncology, and radiation oncology expertise, providing a unified treatment recommendation for each case. This served as the primary clinical baseline.

Framework 2: Multi-Expert Deliberation with Consensus Synthesis. Using a single structured prompt, the model was asked to generate three sequential, independent reasoning paths—one from each specialist perspective — before synthesising a final consensus recommendation. This framework tested whether making the deliberative structure explicit within a single prompt improves recommendation quality through structured internal reasoning, without requiring separate API calls for each role, analogous to how actual tumour boards integrate diverse specialty viewpoints.

Framework 3, 4 and 5: Specialist Role Personas. In each framework, the model was explicitly instructed to assume the role of a surgical oncologist (Framework 3), medical oncologist (Framework 4), or radiation oncologist (Framework 5), and to provide a treatment recommendation, without chain-of-thought scaffolding or other explicit instructions to suppress cross-disciplinary content.

Framework 6: Majority Voting Ensemble. This framework aggregated the three independent role recommendations (Frameworks 3–5) using majority voting: when two or three specialists agreed on the same treatment category, that category became the ensemble recommendation. This framework was included as a methodological comparator to assess whether democratic aggregation adds incremental value beyond individual role performance.

### Treatment categorization and reference standard

Recommendations were mapped to nine mutually exclusive treatmentcategories: (1) best supportive care, (2) further diagnostics, (3) endoscopic resection, (4) active surveillance, (5) localised therapy, (6) systemic therapy, (7) surgery, (8) multimodal therapy, and, (9) multistep therapy.

The actual MDT recommendation served as the reference standard. In cases where the board documented multiple acceptable treatment options, GPT-5 outputs were scored as correct if they matched any valid recommendation.

Inter-rater reliability for the nine-category therapeutic classification was assessed between two board-certified reviewers across all 500 rated responses, resulting in an observed agreement of 93.6% (468/500 cases) and a Cohen’s κ of 0.92, indicating almost perfect agreement per Landis and Koch criteria. Residual disagreements were resolved through discussion until consensus was reached. Annotation criteria and classification guidelines are provided in Supplementary Note 12 and in the public repository.

### Framework comparison

We evaluated concordance between GPT-5 recommendations and MDT decisions, defined as the proportion of cases with concordant treatment categories. Wilson score 95% confidence intervals were used for all proportions to ensure accurate estimation in small subgroups. Overall differences across frameworks were assessed with Cochran’s Q test for repeated binary outcomes; pairwise comparisons used McNemar’s test. Statistical significance was set at α < 0.05.

Bonferroni correction was applied to all pairwise McNemar tests (15 comparisons, corrected α = 0.0033) and to all point-biserial correlation analyses (6 comparisons, corrected α = 0.0083).

Cohen’s kappa coefficients quantified treatment-category concordance between each GPT-5 framework and the MDT reference standard, and between all pairwise framework combinations, interpreted according to Landis and Koch criteria (<0.20 slight; 0.21–0.40 fair; 0.41–0.60 moderate; 0.61–0.80 substantial; >0.80 almost perfect).

### Inter-specialist decision variability and entropy analysis

For each case, inter-specialist variability was quantified by counting the number of unique treatment recommendations across the three role-prompted frameworks (Frameworks 3–5). Shannon entropy (base-2 logarithm) was calculated across these recommendations to formally measure decision uncertainty: values range from 0 bits (complete unanimity) to log₂ (3) ≈ 1.585 bits (maximal three-way disagreement).

Entropy values were analysed descriptively and stratified by tumour diagnosis to characterise inter-specialist decision uncertainty. Inferential modelling of entropy was not performed owing to small subgroup sizes.

### Subgroup and supplementary analyses

Concordance rates were stratified by tumour type, consultation type (initial presentation vs. follow-up), and treatment category to identify clinical contexts where persona performance varied. Subgroup confidence intervals used the Wilson score method. Per-diagnosis confusion matrices were generated for each framework to identify systematic patterns of concordance and discordance.

Role-specific treatment preference profiles were analysed descriptively to characterise differences in treatment framing across specialist roles; this analysis was not intended to assess inter-rater concordance. Results are reported in the Supplementary Material Sections S6 and S7.

### Power analysis

To characterise the statistical sensitivity of pairwise comparisons, a post-hoc power analysis was conducted for all McNemar tests using the observed discordant pair counts. Power was estimated analytically from the proportion of discordant pairs favouring each framework at α = 0.05 (two-sided), using the normal approximation to the McNemar statistic. Results are reported in the Supplementary Material Section S4.

### Role authenticity analysis

To characterise the degree to which GPT-5 adopted specialty-specific reasoning under role-based prompting, we applied four complementary analytical layers: (1) domain-specific content detection, (2) role boundary adherence and cross-disciplinary content analysis, (3) combined content and clinical accuracy analysis, and (4) semantic embedding analysis.

Domain-Specific Content Detection: each role-prompted output was assessed for the presence of at least one specialty-characteristic element, reasoning patterns, or treatment considerations. Surgical oncologist outputs were evaluated for discussion of resectability, operative technique, or perioperative considerations. Medical oncologist outputs were evaluated for reference to systemic therapy regimens, performance status, chemotherapy sequencing or other internal medicine therapies. Radiation oncologist outputs were evaluated for radiation-specific content including dose, fractionation, or normal tissue constraints. The point-biserial correlation between content presence and recommendation concordance was then calculated to test whether authentic role adoption translates to improved clinical accuracy.

Role Boundary Adherence and Cross-Disciplinary Content Analysis: beyond content presence, we assessed whether the model maintained professional role boundaries or generated cross-disciplinary treatment decisions outside its assigned specialty’s domain. Boundary violations were defined as autonomous treatment decisions belonging to another specialty — for example, a surgical oncologist proposing a specific chemotherapy regimen, or a radiation oncologist recommending detailed operative technique. Contextual references to other modalities used solely to frame the prompted specialty’s own recommendation were not classified as violations: a radiation oncologist stating that the tumour has been deemed unresectable by the surgical team appropriately integrates interdisciplinary context without crossing disciplinary boundaries. This distinction between using another domain’s findings as clinical input versus making decisions within that domain formed the basis of all classification decisions. Cross-disciplinary content frequency was calculated as the proportion of role-prompted responses containing boundary violations, stratified by specialty.

Combined Content and Clinical Accuracy Analysis: each role-prompted response was categorised by the joint presence or absence of (i) specialty-specific content and (ii) cross-disciplinary content, resulting in four mutually exclusive categories: role-specific content only, cross-disciplinary content only, both, or neither. For each category, the proportion of recommendations concordant with the MDT decision was calculated per role and then averaged across roles, weighting by subgroup size.

Semantic Embedding Analysis: text embeddings were generated for all GPT-5 outputs using the BAAI/bge-m3 model. Analyses were performed separately for single-prompt specialist persona and for multi-expert single personas to assess structural differences under distinct prompting paradigms.

Embedding-based metrics are interpretive proxies for semantic relatedness and should not be equated with cognitive or reasoning processes.

Pairwise cosine similarity matrices were computed between all specialist roles to quantify semantic proximity.

To characterise the geometric structure of role-specific representations, we computed role centroids in embedding space, pairwise centroid distances, and within-role dispersion (standard deviation along principal axes). Principal component analysis (PCA) was applied to project high-dimensional embeddings into two dimensions for visualisation. PCA analyses were conducted globally across all tumour types and separately within each tumour category.

Distributional differences between role embedding manifolds were further quantified using Jensen–Shannon divergence. This provides a symmetric measure of distributional separation between role-specific semantic manifolds.

To formally test structural separation in embedding space, PCA projections were subjected to Kruskal–Wallis tests across roles for the first two principal components. Effect sizes were estimated using η² (variance decomposition) to quantify the proportion of variance attributable to role assignment.

The choice of non-parametric methods was supported by distributional assumption checks. Of the four principal-component distributions tested, three deviated significantly from normality (Shapiro–Wilk p < 0.001: PC1 and PC2 under specialist persona prompting, and PC1 under multi-expert deliberation); the only component consistent with normality (multi-expert deliberation PC2, *p* = 0.09) nonetheless showed unequal variances across roles (Levene *p* < 0.001). Variance heterogeneity was likewise present for PC1 in both conditions (Levene *p* = 0.008 and *p* = 0.014). Shannon entropy was strongly non-normal (Shapiro–Wilk W = 0.60, *p* < 0.001; 72% of cases at 0 bits) and heteroscedastic across tumour types (Levene *p* < 0.001), and was therefore summarised descriptively rather than modelled inferentially. Non-parametric Kruskal–Wallis tests were thus appropriate for all inferential comparisons of embedding structure; framework-level concordance was compared using distribution-free tests for binary outcomes (Cochran’s Q, McNemar).

Persona drift measures how much a role’s semantic output changes depending on whether it is prompted as a standalone specialist persona or as part of the multi-expert deliberation framework. For each case and role, drift was quantified as the cosine similarity between the two corresponding embeddings — higher similarity indicating less drift. This was summarised at four levels: individual cases, role-level averages, tumour-type subgroups, and role centroids (comparing mean embeddings across the two prompting conditions).

Composite Persona Stability Indices: to provide a multidimensional summary of role-specific behavioural consistency, three composite indices were computed: the Persona Stability Index (PSI), the Composite Robustness Index (CRI), and the Clinical Risk Penalty Score. Index construction, component definitions, theory-driven weight rationale, and sensitivity analysis are described in full in the Supplementary Material Sections S11. Robustness was assessed through weight perturbation sensitivity analysis (±10–20%) and convergent validity between PSI and CRI. All indices should be interpreted as exploratory descriptive summaries of model behaviour within this evaluation framework rather than validated clinical metrics.

### Reproducibility and code availability

The complete analysis pipeline — including preprocessing, statistical testing, embedding analysis, and figure generation — is publicly available at: https://doi.org/10.5281/zenodo.21295353.

The repository includes the complete derived dataset — comprising GPT-5 outputs, treatment category classifications, embedding vectors, and MDT reference labels for all 100 cases — enabling full reproduction of all statistical analyses and figures. Original clinical vignettes are not distributed due to institutional data governance constraints; synthetic cases matching the input schema are provided for prompt pipeline testing. The derived dataset is sufficient for complete reproduction of all reported results.

## Supplementary information

Supplementary Material

## Data Availability

The original pseudonymised datasets generated and/or analysed during the current study are not publicly available in their original form due to institutional data protection policies and patient privacy constraints. A fully anonymised derived dataset—comprising GPT-5 outputs, treatment category classifications, embedding vectors, and MDT reference labels for all 100 cases — is publicly available at: https://doi.org/10.5281/zenodo.21295353. Original clinical vignettes and non-anonymised data may be made available from the corresponding author on reasonable request, subject to institutional approval and applicable data use agreements.

## Code availability

Complete source code, documentation, and detailed implementation specifications are publicly available in the same repository (https://doi.org/10.5281/zenodo.21295353). The repository includes all (pre)processing scripts, prompt templates, and analysis scripts required to reproduce the experimental setup and the reported results. No proprietary software is required beyond standard Python libraries and publicly documented APIs.
