# Supplementary material for: Role prompting modulates linguistic style but not clinical decision structure in GPT-5 tumour board simulation
Source: NPJ Digit Med. 2026 Aug 13;9:626. doi: 10.1038/s41746-026-03069-4 (PMC13473603; doi:10.1038/s41746-026-03069-4)
Supplement: Supplementary file 1 [file 41746_2026_3069_MOESM1_ESM.pdf]

Supplementary Material

Supplementary Note 1 - Prompt Template

Framework 1: Standard Multidisciplinary Tumour Board Simulation

- **System prompt**:

  You are a multidisciplinary oncological Board including surgeons, medical oncologists, radiation oncologists that has to decide the next therapeutic step for the following patient. All patient information is enclosed in triple single quotation marks (''' ''').

- **User prompt**:

  What is your decision as a Tumourboard? If a clinical question (‘Fragestellung’) is present, answer precisely to it. Otherwise, answer based on your knowledge. Since you are the Tumourboard, you cannot suggest presenting the case to a tumourboard or a new multidisciplinary discussion; you must clearly make a decision. Answer in german, in one or two sentences.

Framework 2: Multi-Expert Deliberation with Consensus Synthesis

- **System prompt**:

  You are a multidisciplinary oncological Board including surgeons, medical oncologists, radiation oncologists that has to decide the next therapeutic step for the following patient.

  Use self-consistency reasoning. Generate three independent reasoning paths representing: 1) a surgeon, 2) a medical oncologist, 3) a radiation oncologist. Each path should analyze the following case from that specialty’s perspective and propose management options. After generating the three independent lines of reasoning, synthesize a final consensus recommendation representing the most consistent treatment across specialties.

  Present in German as:

  1. <surgeon>Surgeon reasoning path</surgeon>

  2. <onco>Oncologist reasoning path</onco>

  3. <radioonco>Radiooncologist reasoning path</radioonco>

  4. <tumourboard>Final tumour-board consensus based on self-consistency</tumourboard>.

  All patient information is enclosed in triple single quotation marks (''' ''').

- **User prompt**:

  What is your decision as a Tumourboard? If a clinical question (‘Fragestellung’) is present, answer precisely to it. Otherwise, answer based on your knowledge. Since you are the Tumourboard, you cannot suggest presenting the case to a tumourboard or a new multidisciplinary discussion; you must clearly make a decision. Answer in german, in one or two sentences.

Frameworks 3–5: Single Specialist Role Prompts

- **System prompt**:

  "You are a [role] that has to decide the next therapeutic step for the following patient. All the information about the patient are in triple single quotation marks (''' ''')."

- **User prompt**:

  What is your decision as a [role]? If a clinical question ('Fragestellung') is present answer precisely to it. Otherwise answer based on your knowledge. You can't suggest to present the case to a tumourboard or a new multidisciplinary discussion, you have to clearly make a decision. Answer in german, in one or two sentences, explaining your decision.

Supplementary Note 2 - Demographics

Baseline Characteristics	Overall (N = 100)	Esophageal-Ca (N = 20)	Gastric-Ca (N = 20)	Pancreatic-Ca (N = 20)	Hepatobiliary-Ca (N = 20)	Colorectal-Ca (N = 20)
**Age, Median (IQR)**	64.5 (56-71)	65 (60-70)	67 (61-73)	67 (61-74)	59 (50-70)	62 (54-67)
**Gender**						
**Male, N (%)**	64	15 (75%)	10 (50%)	14 (70%)	13 (65%)	12 (60%)
**Female N (%)**	36	5 (25%)	10 (50%)	6 (30%)	7 (35%)	8 (40%)
**Recommended Therapies by the MDT**						
**Best Supportive Care, N (%)**	1 (1.0%)	-	1 (5.0%)	-	-	-
- FP N (%)	1 (2.0%)	-	1 (10.0%)	-	-	-
- FOLLOW-UP N (%)	-	-	-	-	-	-
**Diagnostic, N (%)**	1 (1.0%)	-	1 (5.0%)	-	-	-
- FP N (%)	-	-	-	-	-	-
- FOLLOW-UP N (%)	1 (2.0%)	-	1 (10.0%)	-	-	-
**Endoscopic Resection, N (%)**	2 (2.0%)	-	2 (10.0%)	-	-	-
- FP N (%)	-	-	-	-	-	-
- FOLLOW-UP N (%)	2 (4.0%)	-	2 (20.0%)	-	-	-
**Active Surveillance, N (%)**	4 (4.0%)	-	1 (5.0%)	-	-	3 (15.0%)
- FP N (%)	1 (2.0%)	-	-	-	-	1 (10.0%)
- FOLLOW-UP N (%)	3 (6.0%)	-	1 (10.0%)	-	-	2 (20.0%)
**Multimodal, N (%)**	7 (7.0%)	7 (35.0%)	-	-	-	-
- FP N (%)	6 (12.0%)	6 (60.0%)	-	-	-	-
- follow-up n (%)	1 (2.0%)	1 (10.0%)	-	-	-	-
**Multistep, N (%)**	19 (19.0%)	3 (15.0%)	6 (30.0%)	1 (5.0%)	1 (5.0%)	8 (40.0%)
- FP N (%)	12 (24.0%)	2 (20.0%)	5 (50.0%)	1 (10.0%)	-	4 (40.0%)
- FOLLOW-UP N (%)	7 (14.0%)	1 (10.0%)	1 (10.0%)	-	1 (10.0%)	4 (40.0%)
**Surgery, N (%)**	29 (29.0%)	3 (15.0%)	5 (25.0%)	11 (55.0%)	7 (35.0%)	3 (15.0%)
- FP N (%)	16 (32.0%)	-	4 (40.0%)	6 (60.0%)	4 (40.0%)	2 (20.0%)
- FOLLOW-UP N (%)	13 (26.0%)	3 (30.0%)	1 (10.0%)	5 (50.0%)	3 (30.0%)	1 (10.0%)
**Systemic Therapy, N (%)**	29 (29.0%)	6 (30.0%)	4 (20.0%)	7 (35.0%)	7 (35.0%)	5 (25.0%)
- FP N (%)	11 (22.0%)	2 (20.0%)	-	3 (30.0%)	4 (40.0%)	2 (20.0%)
- FOLLOW-UP N (%)	18 (36.0%)	4 (40.0%)	4 (40.0%)	4 (40.0%)	3 (30.0%)	3 (30.0%)
**Local Therapy N (%)**	8 (8.0%)	1 (5.0%)	-	1 (5.0%)	5 (25.0%)	1 (5.0%)
- FP N (%)	3 (6.0%)	-	-	-	2 (20.0%)	1 (10.0%)
- FOLLOW-UP N (%)	5 (10.0%)	1 (10.0%)	-	1 (10.0%)	3 (30.0%)	-

Supplementary Table 1. *Patient demographics, baseline clinical characteristics, and tumour board treatment recommendations across tumour subgroups. Values are reported as median (interquartile range) for continuous variables and number (percentage) for categorical variables. Subgroups include patients with esophageal, gastric, pancreatic, hepatobiliary, and colorectal cancers (N = 20 per group). Abbreviations: FP = First Presentation; Ca = Cancer, MDT = Multidisciplinary Tumourboard*

Supplementary Note 3 - Framework Comparison

Comparison	b	c	χ^2	Raw p	Bonferroni p
Sim. Tumour Board vs Multi-expert Delib.	5	4	0.000	1.000	1.000
Sim. Tumour Board vs Surgical Oncologist	5	6	0.000	1.000	1.000
Sim. Tumour Board vs Medical Oncologist	3	4	0.000	1.000	1.000
Sim. Tumour Board vs Radiation Oncologist	10	5	1.067	0.302	1.000
Sim. Tumour Board vs Majority Vote	3	7	0.900	0.343	1.000
Multi-expert Delib. vs Surgical Oncologist	6	8	0.071	0.789	1.000
Multi-expert Delib. vs Medical Oncologist	4	6	0.100	0.752	1.000
Multi-expert Delib. vs Radiation Oncologist	9	5	0.643	0.423	1.000
Multi-expert Delib. vs Majority Vote	3	8	1.455	0.228	1.000
Surgical Oncologist vs Medical Oncologist	5	5	0.100	0.752	1.000
Surgical Oncologist vs Radiation Oncologist	9	3	2.083	0.149	1.000
Surgical Oncologist vs Majority Vote	0	3	1.333	0.248	1.000
Medical Oncologist vs Radiation Oncologist	10	4	1.786	0.181	1.000
Medical Oncologist vs Majority Vote	2	5	0.571	0.450	1.000
Radiation Oncologist vs Majority Vote	0	9	7.111	0.008	0.115

Supplementary Table 2. *Pairwise McNemar tests. Each row represents one pairwise comparison between frameworks. b = number of cases where the first framework was concordant with the MDT recommendation and the second was not; c = number of cases where the second framework was concordant and the first was not. χ^2 = McNemar test statistic. Bonferroni p = raw p-value multiplied by the number of comparisons (n = 15), capped at 1.000; corrected $\alpha = 0.0033$. No comparison survived Bonferroni correction. Bold row indicates the only comparison reaching nominal statistical significance ($p < 0.05$). Sim. = Simulated; Delib. = Deliberation. n = 100 cases per framework.*

Supplementary Note 4 - Post-hoc McNemar Power Analysis

Post-hoc power was estimated analytically for all 15 pairwise McNemar comparisons using observed discordant pair counts. Power ranged from 0.05 (surgeon vs oncologist: n_discordant = 10, symmetric discordance) to 0.85 (radiation oncologist vs majority vote: n_discordant = 9, all cases favouring majority vote). The majority of comparisons returned power below 0.15, reflecting small numbers of discordant cases rather than large undetected differences. Notably, b = 0 for both majority vote vs surgeon and majority vote vs radiation oncologist comparisons, indicating directional dominance of the ensemble in all discordant cases for these pairs. Low overall power is consistent with the high inter-framework concordance reported in the main text (κ = 0.724–0.898); when frameworks agree on the large majority of cases, the McNemar test has limited discriminative capacity by design.

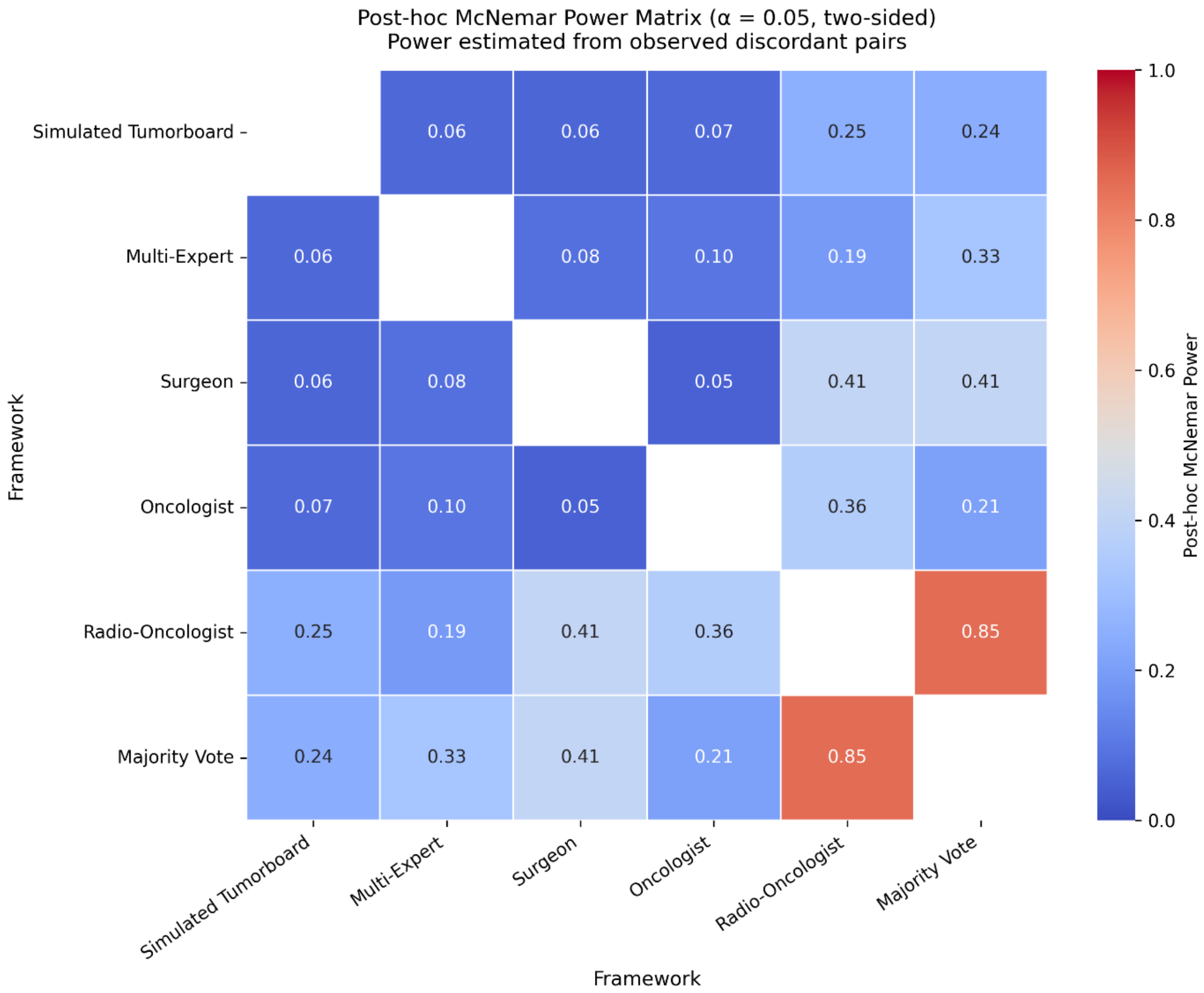

Supplementary Figure 1. *Post-hoc McNemar Power Matrix heatmap.*

model_1	model_2	b (col1 correct, col2 wrong)	c (col1 wrong, col2 correct)	n_discordant	effect_size (p_disc)	post_hoc_power
Simulated Tumourboard	Multi-Expert	5	4	9	0,556	0,063
Simulated Tumourboard	Surgeon	5	6	11	0,455	0,061
Simulated Tumourboard	Oncologist	3	4	7	0,429	0,067
Simulated Tumourboard	Radio-Oncologist	10	5	15	0,667	0,252
Simulated Tumourboard	Majority Vote	3	7	10	0,300	0,244
Multi-Expert	Surgeon	6	8	14	0,429	0,083
Multi-Expert	Oncologist	4	6	10	0,400	0,097
Multi-Expert	Radio-Oncologist	9	5	14	0,643	0,188
Multi-Expert	Majority Vote	3	8	11	0,273	0,326
Surgeon	Oncologist	5	5	10	0,500	0,050
Surgeon	Radio-Oncologist	9	3	12	0,750	0,410
Surgeon	Majority Vote	0	3	3	0,000	0,410
Oncologist	Radio-Oncologist	10	4	14	0,714	0,361
Oncologist	Majority Vote	2	5	7	0,286	0,205
Radio-Oncologist	Majority Vote	0	9	9	0,000	0,851

Supplementary Table 3. *Pairwise discordant counts, effect sizes, and post-hoc power for all 15 comparisons.*

Supplementary Note 5 - Inter-Framework Agreement

Cohen's kappa coefficients between GPT-5 frameworks and the MDT reference standard ranged from κ = 0.612 (radiation oncologist) to κ = 0.720 (surgical oncologist), indicating substantial agreement throughout (Landis & Koch criteria). The simulated tumour board achieved κ = 0.670, multi-expert deliberation κ = 0.701, and the majority vote ensemble κ = 0.708 (Supplementary Figure 2).

Inter-framework agreement among all GPT-5 frameworks ranged from substantial to almost perfect (κ = 0.672–0.898). Among specialist persona frameworks, inter-role kappa ranged from κ = 0.724 (surgical vs radiation oncologist) to κ = 0.898 (surgical oncologist vs majority vote ensemble), with the latter reaching almost perfect agreement. The simulated tumour board showed strongest agreement with the medical oncologist persona (κ = 0.820, almost perfect) and weakest with the radiation oncologist (κ = 0.672, substantial), mirroring the pattern observed against the MDT reference.

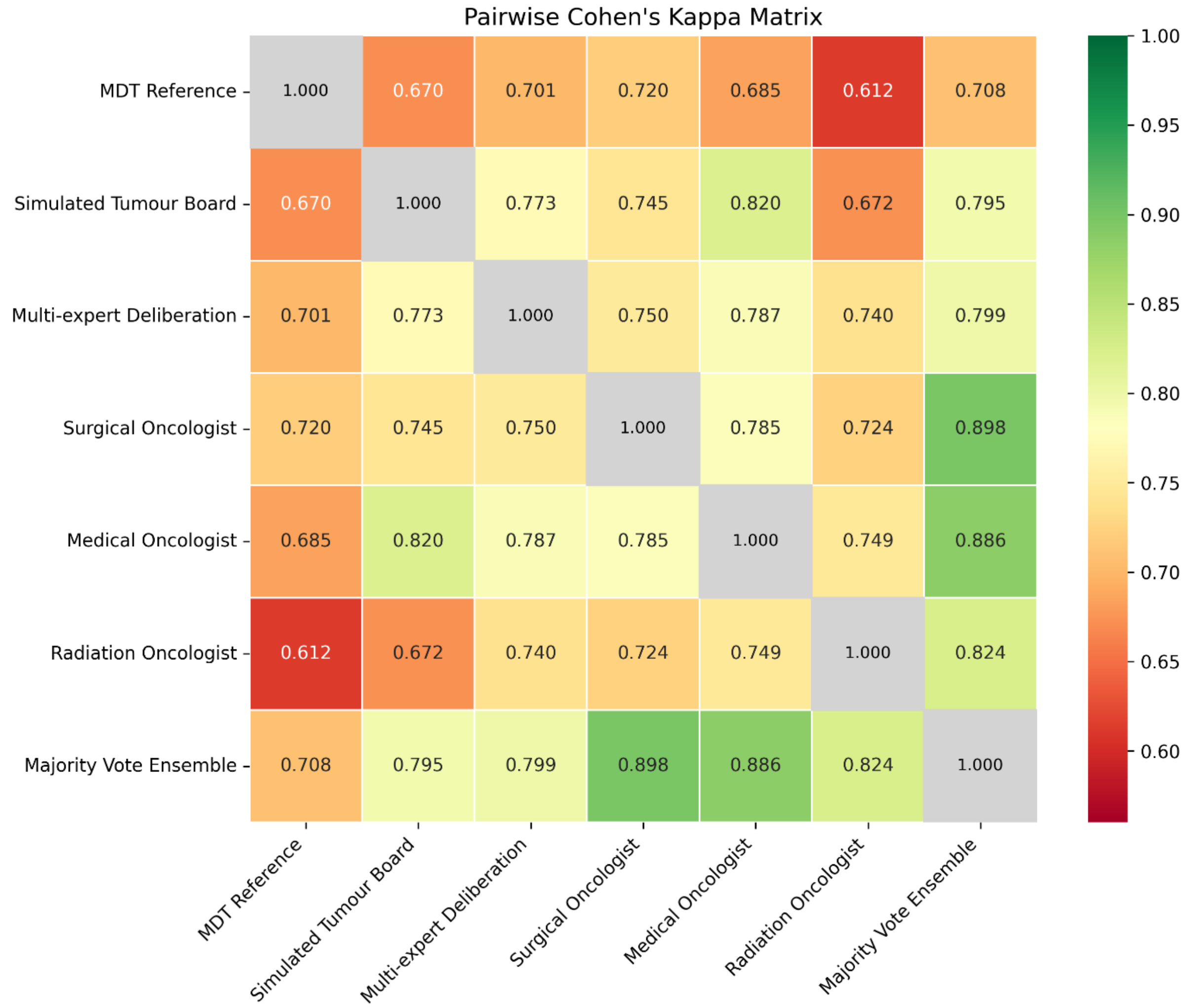

Supplementary Figure 2. *Pairwise Cohen's kappa matrix across all frameworks and the MDT reference standard. Each cell represents the kappa coefficient between the corresponding pair of methods. The diagonal (grey) represents perfect self-agreement (κ = 1.000). Colour scaling ranges from the observed minimum (κ = 0.612, red) to perfect agreement (κ = 1.000, green), restricted to the empirical data range to maximise visual contrast. Abbreviations: MDT = multidisciplinary tumour board; Sim. TB = simulated tumour board (Framework 1); Multi-exp. = multi-expert deliberation (Framework 2); Surg. = surgical oncologist (Framework 3); Med. = medical oncologist (Framework 4); Rad. = radiation oncologist*

(Framework 5); MV = majority vote ensemble (Framework 6). All values are interpreted according to Landis and Koch criteria (<0.20 slight; 0.21–0.40 fair; 0.41–0.60 moderate; 0.61–0.80 substantial; >0.80 almost perfect).

Supplementary Note 6 - Performance Stratified by Tumour Type and Consultation Context

Concordance rates varied substantially across tumour types (Supplementary Figure 3). Oesophageal cancer demonstrated the highest concordance, with surgical oncologist, medical oncologist, and majority vote all achieving 100% concordance (20/20; 95% CI: 83.9%–100.0%), and the remaining frameworks reaching 95% (19/20; 95% CI: 76.4%–99.1%). Pancreatic cancer showed 90% concordance for surgical oncologist, radiation oncologist, and majority vote (18/20; 95% CI: 69.9%–97.2%) versus 80% for simulated tumour board and medical oncologist (16/20; 95% CI: 58.4%–91.9%). Gastric cancer exhibited the widest intra-tumour range: medical oncologist reached 95% (19/20) while radiation oncologist achieved 70% (14/20; 95% CI: 48.1%–85.5%). Colorectal cancer showed the most variable and overall lowest performance (65%–80%), consistent with its highest entropy. Hepatobiliary cancer ranged from 60% (radiation oncologist) to 90% (surgical oncologist and majority vote).

Concordance rates were comparable between initial presentations (76%–86% across frameworks) and follow-up consultations (80%–88%) (Supplementary Figure 4).

Tumour Type	Framework	N	Correct	Concordance (%)	CI lower	CI upper
Oesophageal	Sim. Tumour Board	20	19	95.0	76.4%	99.1%
	Multi-expert Delib.	20	19	95.0	76.4%	99.1%
	Surgical Oncologist	20	20	100.0	83.9%	100.0%
	Medical Oncologist	20	20	100.0	83.9%	100.0%
	Radiation Oncologist	20	19	95.0	76.4%	99.1%
	Majority Vote	20	20	100.0	83.9%	100.0%
Pancreatic	Sim. Tumour Board	20	16	80.0	58.4%	91.9%
	Multi-expert Delib.	20	16	80.0	58.4%	91.9%
	Surgical Oncologist	20	18	90.0	69.9%	97.2%
	Medical Oncologist	20	16	80.0	58.4%	91.9%
	Radiation Oncologist	20	18	90.0	69.9%	97.2%
	Majority Vote	20	18	90.0	69.9%	97.2%
Gastric	Sim. Tumour Board	20	18	90.0	69.9%	97.2%
	Multi-expert Delib.	20	15	75.0	53.1%	88.8%
	Surgical Oncologist	20	17	85.0	64.0%	94.8%
	Medical Oncologist	20	19	95.0	76.4%	99.1%
	Radiation Oncologist	20	14	70.0	48.1%	85.5%
	Majority Vote	20	17	85.0	64.0%	94.8%
Colorectal	Sim. Tumour Board	20	14	70.0	48.1%	85.5%
	Multi-expert Delib.	20	16	80.0	58.4%	91.9%
	Surgical Oncologist	20	13	65.0	43.3%	81.9%
	Medical Oncologist	20	16	80.0	58.4%	91.9%
	Radiation Oncologist	20	15	75.0	53.1%	88.8%
	Majority Vote	20	16	80.0	58.4%	91.9%
Hepatobiliary	Sim. Tumour Board	20	16	80.0	58.4%	91.9%
	Multi-expert Delib.	20	16	80.0	58.4%	91.9%
	Surgical Oncologist	20	16	80.0	58.4%	91.9%
	Medical Oncologist	20	13	65.0	43.3%	81.9%
	Radiation Oncologist	20	12	60.0	38.7%	78.1%
	Majority Vote	20	16	80.0	58.4%	91.9%

Supplementary Table 4*: Concordance rates stratified by tumour type — Wilson 95% confidence intervals.*

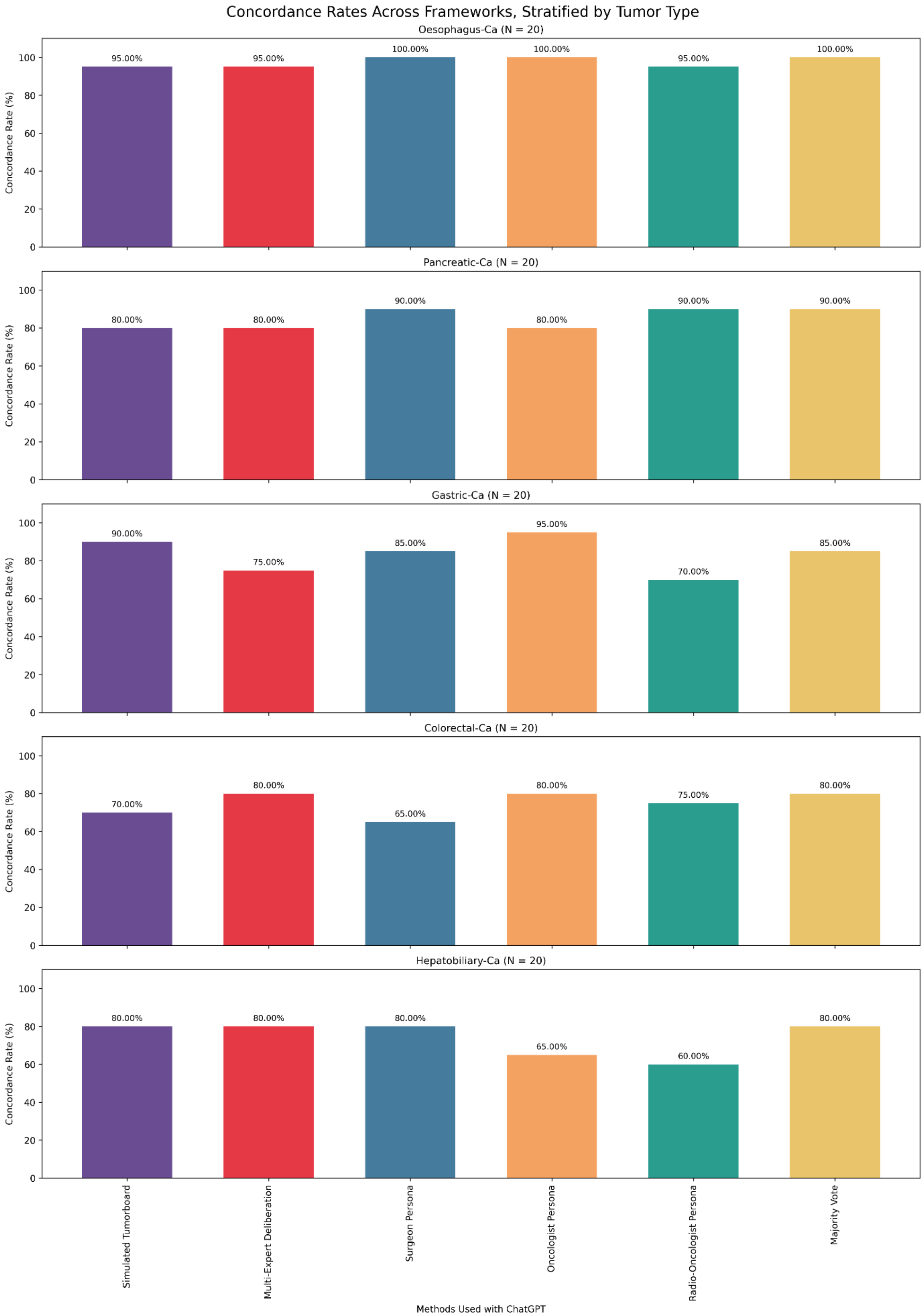

Supplementary Figure 3. *Overall concordance rate across 6 framework configurations, stratified by tumour diagnosis. Each concordant or non-concordant answer accounts for 5 percentage points.*

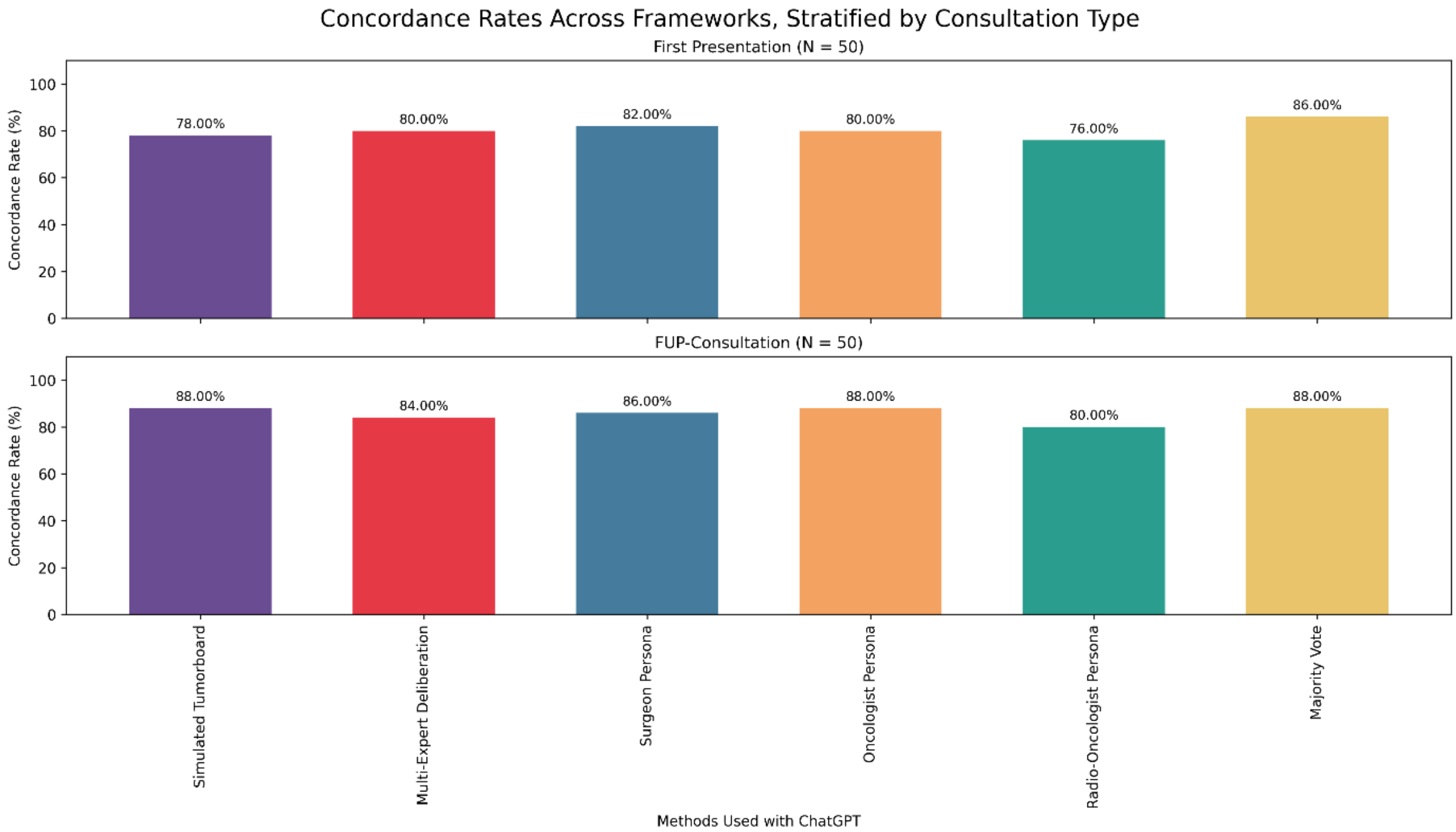

Supplementary Figure 4. *Overall concordance rates across 6 framework configurations stratified by consultation type (initial presentation vs follow-up). Each concordant or non-concordant answer accounts for 2 percentage points.*

Supplementary Note 7 - Performance by Treatment Category

Framework performance varied substantially across treatment categories (Supplementary Figure 5). High-concordance categories (>89% concordance) included multistep therapy (17–18/19 cases), multimodal therapy (6–7/7 cases), and systemic therapy (24–29/29 cases). Best supportive care showed perfect concordance (1/1 case) with zero inter-specialist variability. Surgery recommendations showed the most framework-dependent performance (59%–76% concordance; mean variability: 1.55 unique recommendations per case), with the surgical oncologist framework not producing the highest surgical concordance — underscoring the multi-factorial nature of resectability decisions. Active surveillance demonstrated 50–75% concordance across only 4 cases, precluding reliable interpretation.

Mean recommendation variability by treatment category: best supportive care (1.00), active surveillance (1.25), systemic therapy (1.28), multimodal therapy (1.43), multistep therapy (1.53), surgery (1.55).

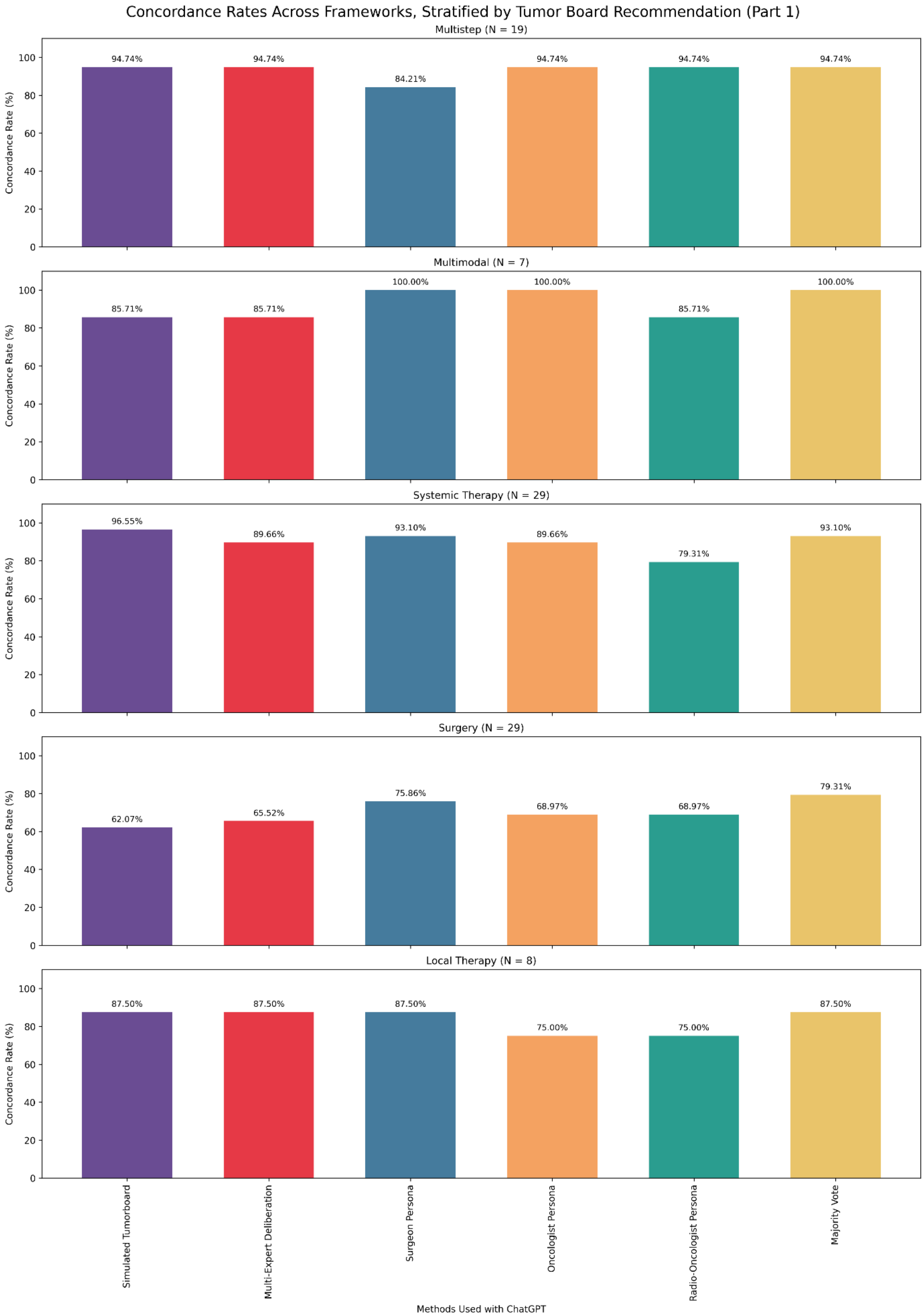

Concordance Rates Across Frameworks, Stratified by Tumor Board Recommendation (Part 1)
Multistep (N = 19)
94.74%
94.74%
84.21%
94.74%
94.74%
94.74%
Multimodal (N = 7)
85.71%
85.71%
100.00%
100.00%
85.71%
100.00%
Systemic Therapy (N = 29)
96.55%
89.66%
93.10%
89.66%
79.31%
93.10%
Surgery (N = 29)
62.07%
65.52%
75.86%
68.97%
68.97%
79.31%
Local Therapy (N = 8)
87.50%
87.50%
87.50%
75.00%
75.00%
87.50%
Concordance Rate (%)
0
20
40
60
80
100
Simulated Tumorboard
Multi-Expert Deliberation
Surgeon Persona
Oncologist Persona
Radio-Oncologist Persona
Majority Vote
Methods Used with ChatGPT

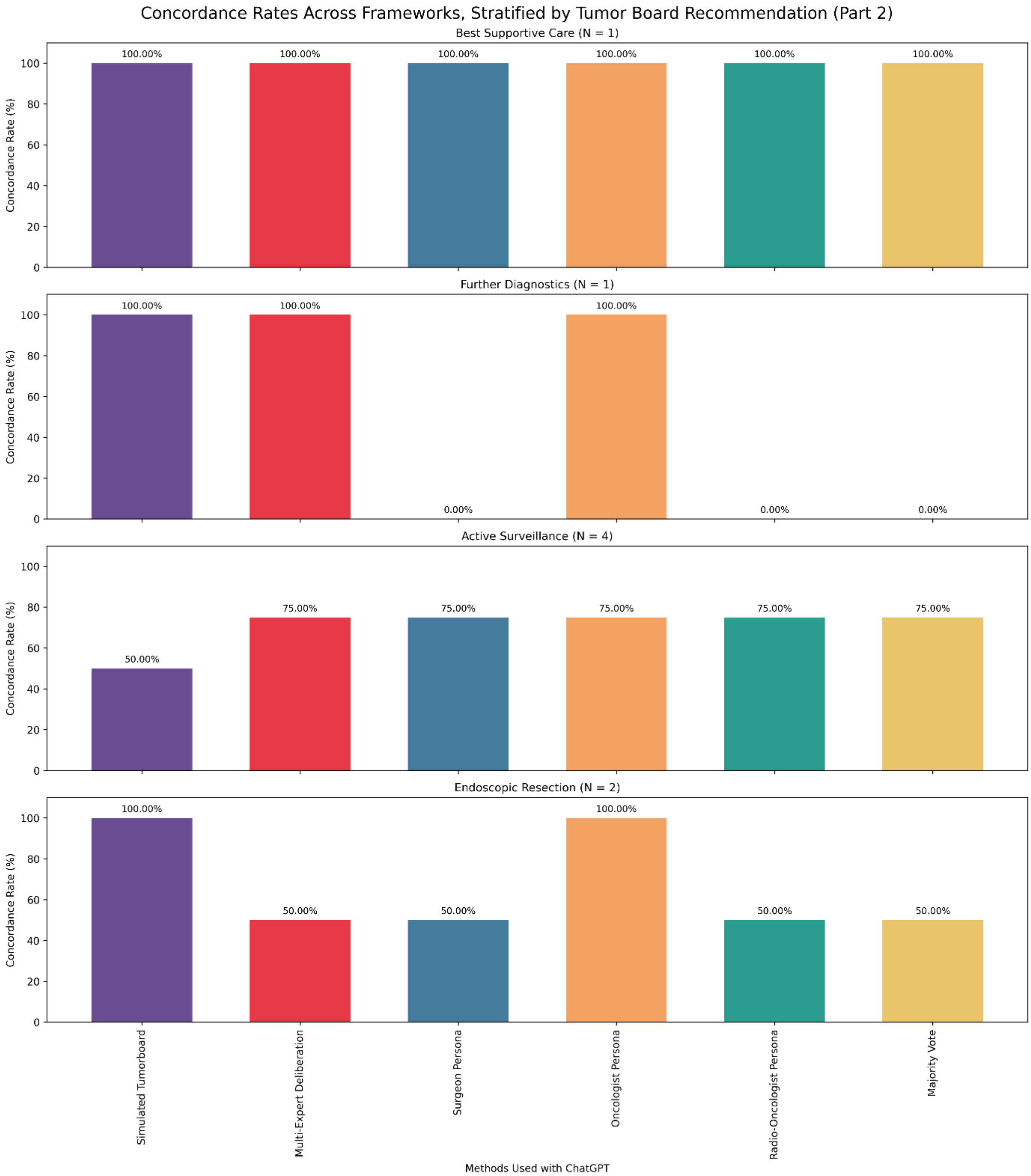

***Supplementary Figure 5 Part 1 and Part 2**: Concordance rates stratified by primary treatment category. Overall concordance rate across 6 framework configurations, stratified by therapeutic recommendation. Each concordant or non-concordant answer contributes proportionally to the total concordance rate, with impact per answer calculated as 100% divided by the number of cases (N) for each therapy category: multistep 5.26%, multimodal 14.29%, systemic therapy 3.45%, surgery 3.45%, local therapy 12.5%, best supportive care 100%, further diagnostics 100%, active surveillance 25%, and endoscopic resection 50%.*

Supplementary Note 8 - Role Authenticity Analysis

Category	Specialist Persona Prompting				Multi-Expert Deliberation			
	Distribution, n (%)				Distribution, n (%)			
	Surgeon (n=100)	Oncologist (n=100)	Radiation Oncologist (n=100)	Overall (n=300)	Surgeon (n=100)	Oncologist (n=100)	Radiation Oncologist (n=100)	Overall (n=300)
Role-specific only	38 (38.0%)	61 (61.0%)	44 (44.0%)	143 (47.7%)	80 (80.0%)	93 (93.0%)	94 (94.0%)	267 (89.0%)
Cross-disciplinary only	5 (5.0%)	1 (1.0%)	1 (1.0%)	7 (2.3%)	0 (0.0%)	0 (0.0%)	0 (0.0%)	0 (0.0%)
Both role-specific and cross-disciplinary	56 (56.0%)	37 (37.0%)	55 (55.0%)	148 (49.3%)	20 (20.0%)	7 (7.0%)	6 (6.0%)	33 (11.0%)
Neither	1 (1.0%)	1 (1.0%)	0 (0.0%)	2 (0.7%)	0 (0.0%)	0 (0.0%)	0 (0.0%)	0 (0.0%)

Supplementary Table 5. *Distribution of GPT-5 responses by content category, stratified by specialist role and prompting framework. Values indicate the number of responses (n) and column-wise percentage (%) within each content category, separately for specialist persona prompting (Frameworks 3–5, n = 300) and multi-expert deliberation (Framework 2, n = 300). Categories are mutually exclusive: role-specific content refers to presence of specialty-characteristic clinical terminology; cross-disciplinary content refers to treatment recommendations outside the assigned specialty scope (boundary violation). Overall represents pooled counts across all three roles.*

Supplementary Note 9 - Tumour-Type-Stratified Embedding Analysis and Confusion Matrices

Tumour-Type-Specific PCA

In the specialist persona setting, colorectal cancer exhibited the greatest role dispersion and the largest surgeon–tumour board centroid displacement (0.126), whereas gastric and hepatobiliary cancers showed tighter clustering. Under multi-expert deliberation, centroid separation increased across all tumour types: oncologist–surgeon separation reached 0.316 (oesophageal), 0.331 (pancreatic), 0.292 (gastric), 0.362 (colorectal), and 0.344 (hepatobiliary), suggesting structurally reinforced role specialisation under deliberative prompting (Supplementary Table 8).

Tumour Type	Role	PC1 centroid	PC2 centroid	Dist. to TB centroid	PC1 dispersion (SD)
Oesophageal	Oncologist	0.098	−0.087	0.024	0.124
	Radio-Oncologist	0.089	−0.108	0.040	0.086
	Surgeon	0.005	−0.066	0.079	0.116
Pancreatic	Oncologist	−0.048	0.005	0.033	0.191
	Radio-Oncologist	−0.020	−0.015	0.014	0.136
	Surgeon	−0.101	0.012	0.087	0.131
Gastric	Oncologist	−0.043	−0.074	0.006	0.163
	Radio-Oncologist	−0.036	−0.095	0.026	0.113
	Surgeon	−0.098	−0.098	0.056	0.157
Colorectal	Oncologist	0.077	0.089	0.032	0.145
	Radio-Oncologist	0.014	0.021	0.092	0.120
	Surgeon	−0.030	0.074	0.126	0.131
Hepatobiliary	Oncologist	0.021	0.112	0.001	0.140
	Radio-Oncologist	−0.016	0.098	0.040	0.130
	Surgeon	−0.051	0.098	0.074	0.149

Supplementary Table 6. *Tumour-type-stratified PCA centroid coordinates and dispersion for specialist persona roles relative to the simulated tumour board. PC1 and PC2 centroid values represent the mean projection of each role's output embeddings onto the first two principal components derived from the full specialist persona embedding space. Distance to tumour board (TB) centroid is the Euclidean distance in the PC1–PC2 plane between each specialist role centroid and the corresponding simulated tumour board centroid, computed separately per tumour type. PC1 dispersion (SD) reflects the standard deviation of individual case projections onto PC1, indexing within-role output variability. Higher centroid distances indicate greater representational divergence from the tumour board reference; higher dispersion values indicate less consistent role-specific outputs across cases.*

Confusion Matrices

Confusion matrices for each tumour type (oesophageal, pancreatic, gastric, colorectal, hepatobiliary) and each framework (surgical oncologist, medical oncologist, radiation oncologist, majority vote) are provided as separate Excel worksheets in the repository (see below). Key patterns are summarised below.

Oesophageal: Near-perfect concordance across all frameworks. The surgical oncologist and medical oncologist each produced one discordant case (multimodal therapy misclassified as systemic therapy). The radiation oncologist produced one discordant case in which the MDT recommended multimodal therapy but best supportive care was recommended.

Pancreatic: Primary discordance involved misclassification of MDT surgery recommendations as non-operative approaches. The simulated tumour board and medical oncologist each misclassified 2 surgery cases as follow-up/active surveillance. The medical oncologist additionally misclassified 1 surgery case each as multimodal and multistep therapy, contributing to its lower overall concordance (80%) compared to surgical oncologist, radiation oncologist, and majority vote (all 90%).

Gastric: Widest inter-role concordance range. The medical oncologist achieved near-perfect concordance (19/20, 95%), with errors limited to one surgery case misclassified as diagnostics. The radiation oncologist showed the most discordant pattern (14/20, 70%): surgery cases were misclassified as endoscopic resection (n=1) and local therapy (n=1); systemic therapy cases were misclassified as multimodal therapy (n=2); and follow-up cases as active surveillance misclassified (n=1). The simulated tumour board misclassified 2 surgery cases as diagnostics.

Colorectal: Most challenging tumour type across all frameworks. The dominant error pattern was confusion between multistep therapy and systemic therapy — a clinically meaningful distinction involving sequential staging-directed treatment versus palliative cytotoxic intent. The surgical oncologist showed the highest error rate (13/20, 65%), with 3 multistep cases misclassified as systemic therapy. The radiation oncologist and medical oncologist each had 4 multistep cases misclassified as systemic therapy. Majority voting partially attenuated individual role biases but could not fully resolve this ambiguity.

Hepatobiliary: Highest inter-specialist entropy. The radiation oncologist showed the most divergent error profile: 3 systemic therapy cases were misclassified as multimodal therapy, and 1 surgery case each was misclassified as multimodal therapy, systemic therapy, and further diagnostics. The medical oncologist misclassified 3 surgery cases as systemic therapy and 1 local therapy case as follow-up. The surgical oncologist and majority vote achieved the highest concordance (16/20, 80%), with errors concentrated in surgery cases misclassified as systemic therapy (n=1 each) and one best supportive care recommendation in a surgery case (surgeon only).

NOTE: Confusion matrices stratified by tumour type and framework. *Full confusion matrices for all tumour type–framework combinations can be reproduced from the publicly available analysis code using the script* 04_advanced_analysis.py *(Section A), with outputs written to* output/advanced_analysis/confusion_matrices_all.xlsx.

Supplementary Note 10 - Full Embedding Geometry — Jensen–Shannon Divergence, Centroid Separation, and Persona Drift

Jensen–Shannon Divergence

Specialist persona condition: JSD ranged from 0.0176 (surgeon vs oncologist) to 0.0969 (simulated tumour board vs surgeon). Multi-expert deliberation condition: JSD ranged from 0.0182 (simulated tumour board vs oncologist) to 0.0932 (surgeon vs oncologist). Distributional divergence magnitudes were comparable across prompting strategies; role pairings of maximal divergence shifted between conditions.

Role Pair		Jensen–Shannon Divergence	
Role A	Role B	Specialist Persona (Frameworks 3–5)	Multi-Expert Deliberation (Framework 2)
Simulated Tumour Board	Surgeon	0,0969	0,0818
Simulated Tumour Board	Oncologist	0,0826	0,0182
Simulated Tumour Board	Radiation Oncologist	0,0357	0,0729
Surgeon	Oncologist	0,0176	0,0932
Surgeon	Radiation Oncologist	0,0646	0,0363
Oncologist	Radiation Oncologist	0,0502	0,0831

Supplementary Table 7. *Pairwise Jensen–Shannon Divergence (JSD) between role-specific embedding distributions under specialist persona prompting (Frameworks 3–5) and multi-expert deliberation (Framework 2). JSD values range from 0 (identical distributions) to 1 (maximally divergent). Highlighted cells (bold) indicate the minimum and maximum values within each condition. Values are reported to four decimal places.*

Persona Drift Statistics

Case-level cosine similarity between specialist persona and multi-expert deliberation embeddings: surgeon 0.797 ± 0.061; simulated tumour board 0.792 ± 0.054; oncologist 0.758 ± 0.070; radiation oncologist 0.750 ± 0.063. Centroid-level drift was minimal across all roles (simulated tumour board: 0.958; surgeon: 0.980; oncologist: 0.974; radiation oncologist: 0.971), indicating stable aggregate role representations despite case-level variability.

Role-Specific Treatment Preference Profiles

Treatment recommendation distributions differed modestly but characteristically across specialist roles (Supplementary Figure 6). Systemic therapy was the most frequently recommended modality across all roles (medical oncologist: 35%; surgical oncologist: 32%; radiation oncologist: 30%). Surgical

intervention was recommended in 25% of surgical oncologist cases, 23% of radiation oncologist cases, and 21% of medical oncologist cases. Multimodal approaches were more prevalent in radiation oncologist (15%) and medical oncologist (12%) outputs versus surgical oncologist (9%), consistent with radiotherapy's natural integration into combined-modality strategies.

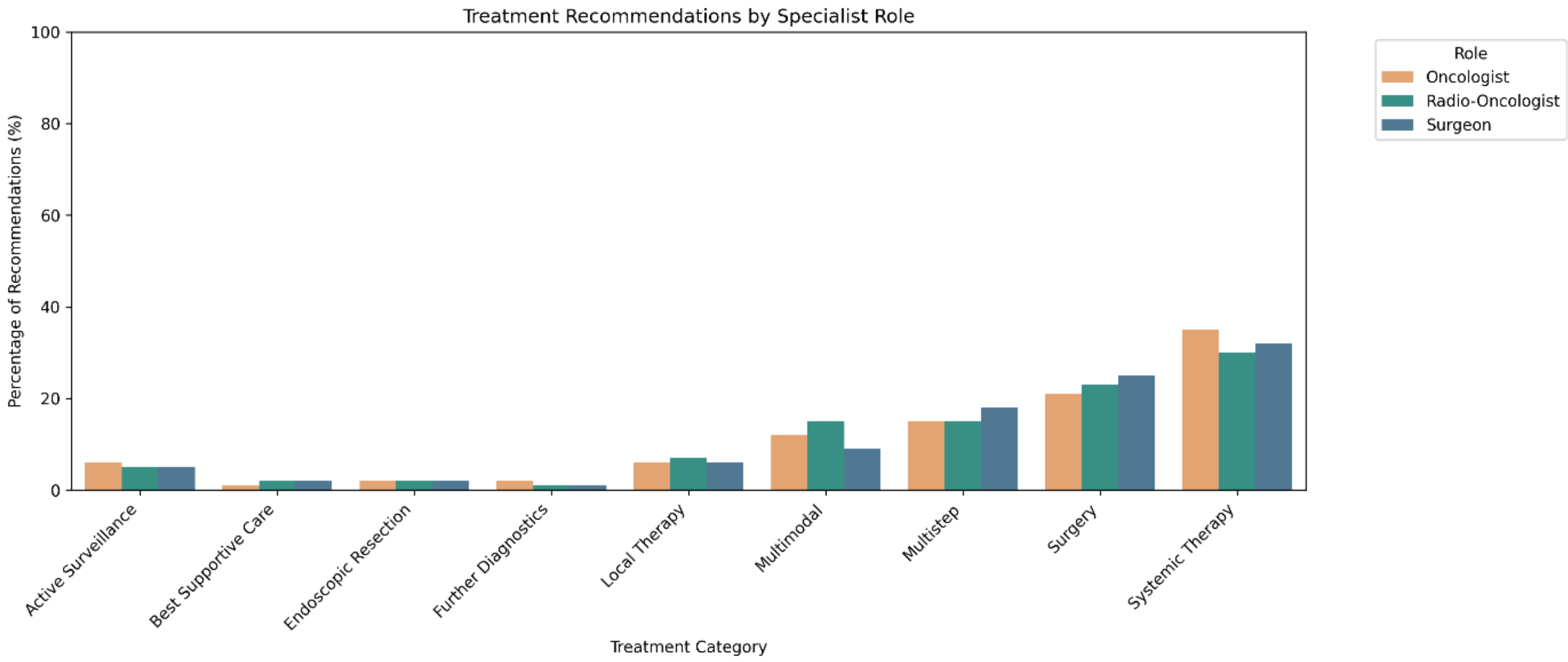

Supplementary Figure 6. *Treatment recommendations by specialist role. Bars show the proportion of each treatment category recommended by surgeons, medical oncologists, and radiation oncologists, highlighting role-specific preference patterns.*

Supplementary Note 11 - Composite Persona Stability Indices — Construction, Rationale, and Sensitivity Analysis

Overview and Epistemological Status

The Persona Stability Index (PSI), Composite Robustness Index (CRI), and Clinical Risk Penalty Score are exploratory composite measures introduced in this study to summarise multi-dimensional behavioural patterns across prompting conditions. These indices have not been externally validated against independent cohorts or clinical outcomes and should be interpreted as hypothesis-generating descriptive tools rather than clinically validated metrics. They are not intended as generalizable performance measures beyond this evaluation framework.

PSI Construction

The PSI integrates four components: (1) semantic identity preservation, quantified as mean cosine similarity between specialist persona and multi-expert deliberation embeddings; (2) role-specific clinical content presence (domain specificity rate); (3) boundary control, defined as 1 − boundary violation rate; and (4) treatment recommendation accuracy. Components are linearly aggregated using theory-driven weights centralised in config.py of the public repository. No data-driven weight optimisation was performed. The formula is:

$PSI = w_1 \cdot cosine\_similarity + w_2 \cdot specificity\_rate + w_3 \cdot (1 - boundary\_violation\_rate) + w_4 \cdot accuracy$

CRI Construction

$CRI = PSI\_components + w_5 \cdot (1 - mean\_treatment\_entropy)$. The entropy stability term penalises personas whose treatment recommendations exhibit high distributional unpredictability across cases.

Clinical Risk Penalty Score

$CRP = w\_boundary \cdot boundary\_violation\_rate + w\_nonspec \cdot (1 - specificity\_rate)$.

Full Numerical Results

Role	PSI	CRI	CRI CI 2.5%	CRI CI 97.5%	Clinical Risk Score	Boundary Entropy (bits)
Surgical Oncologist	0.763	0.768	0.713	0.807	0.390	0.965
Medical Oncologist	0.805	0.810	0.757	0.854	0.236	0.958
Radiation Oncologist	0.763	0.765	0.711	0.808	0.340	0.990

Supplementary Table 8. *Composite persona stability indices per specialist role. PSI, Persona Stability Index; CRI, Composite Robustness Index; CI, confidence interval. The PSI integrates semantic identity preservation (embedding cosine similarity), role-specific clinical content presence, boundary control (1 − boundary violation rate), and treatment recommendation accuracy using theory-driven weights (see Supplementary Note 2 for full construction details). The CRI extends the PSI with a global entropy stability penalty term. Bootstrap 95% CIs were computed for the CRI using 1,000 resamples. The Clinical Risk Penalty Score combines boundary violation rate (weight 0.60) and non-specificity rate (weight*

0.40). The Simulated Tumour Board framework is excluded from PSI and Clinical Risk Penalty Score interpretation as specialty-boundary metrics (specificity rate, boundary control) are not applicable to a non-role-specific prompting condition. All indices are exploratory composite measures that have not been externally validated; values should be interpreted as descriptive summaries of model behaviour within this evaluation framework.

Sensitivity Analysis

PSI scores were recomputed under systematic weight perturbations of ±10% and ±20%, with renormalisation after each perturbation. Role rank ordering was invariant across all perturbation levels (Supplementary Table 9), and Spearman rank correlation between baseline and perturbed scores was 1.000 at every perturbation level — indicating complete rank stability. Component contribution analysis showed that boundary control accounted for 58.6% of PSI score variance, followed by accuracy (34.6%) and specificity rate (6.8%); no single component dominated at the expense of the others, though boundary control was the largest single contributor. For CRI, the distribution was nearly identical (boundary control 58.6%, accuracy 34.6%, specificity rate 6.8%), with entropy stability contributing negligibly (0.02%), indicating that this term adds minimal discriminative variance in the current dataset.

**Perturbation**	**Medical Oncologist**	**Radiation Oncologist**	**Surgical Oncologist**	**Simulated Tumourboard**
-20%	1	2	3	4
-10%	1	2	3	4
0% (baseline)	1	2	3	4
+10%	1	2	3	4
+20%	1	2	3	4

***Supplementary Table 9**. PSI rank stability under weight perturbation (±10%, ±20%). Rank position 1 = highest PSI. Perturbations applied uniformly to all PSI component weights with renormalisation. The Simulated Tumour Board is included for completeness but excluded from specialist role comparisons (see Supplementary Note 2).*

Technical note: In the public repository (03_persona_stability_analysis.py), the sensitivity analysis uses a fixed cosine similarity value of 0.8 to avoid recomputing embeddings for each perturbation step. As this value is identical across roles, it does not influence inter-role rank ordering. Full embedding recomputation is used for all primary PSI and CRI values reported in the manuscript.

Supplementary Note 12 — Annotation Guidelines

Detailed annotation criteria, decision rules, illustrative examples, and the complete annotation workflow are provided in the public repository (prompts/annotation.md; https://doi.org/10.5281/zenodo.21295353).

Category	Definition & Key Criteria
**1. Best Supportive Care**	Palliative or symptom-oriented management only, without intent to modify tumor progression. Includes local stent placement for symptom relief.
**2. Further Diagnostic Procedures**	Standalone diagnostic measures recommended **without a planned therapy**. Examples: histological confirmation, endoscopy, EUS-guided biopsy, diagnostic laparoscopy. **If a therapeutic plan is outlined after diagnostics** (i.e. diagnostic + systemic therapy/surgery), **classify according to that therapy.**
**3. Endoscopic Intervention**	Therapeutic endoscopic procedures, e.g., endoscopic resection or full-thickness endoscopic excision. **Excludes purely diagnostic endoscopy.**
**4. Active Surveillance / Follow-up**	Ongoing monitoring or imaging without active therapy. Includes routine follow-up after curative surgery if no adjuvant therapy is planned.
**5. Multimodal Therapy**	Concurrent use of multiple treatment modalities. Example: radiochemotherapy, chemoradiation. **Only concurrent treatments**; sequential therapies are classified as Multistep.
**6. Multistep Therapy**	Sequential treatment strategies where multiple interventions are planned **before initiation of therapy**. Example: neoadjuvant chemotherapy → surgery → (adjuvant therapy).<br>**Rules:**<br>- If therapy already started, classify based on current stage (e.g., surgery or systemic therapy).<br>- **If tumor resectability is uncertain or only hypothetical, classify the recommendation according to the intended therapy rather than as Multistep.** *For example, if systemic therapy is given to see whether the tumor shrinks enough to become resectable, classify it as Systemic Therapy, not Multistep.*<br>- Exception: a diagnostic laparoscopy performed with planned subsequent systemic therapy (e.g., laparoscopy + port placement followed by chemotherapy) is classified as Multistep Therapy.
**7. Surgery**	**Invasive procedures requiring general anesthesia, including transplantation.** Excludes purely diagnostic laparoscopy (i.e., laparoscopy performed solely for diagnosis, without a planned therapeutic intervention; if surgery is decided intraoperatively based on exploration findings, classify as Surgery).
**8. Systemic Therapy**	Systemic anti-cancer therapy, including chemotherapy, immunotherapy, targeted therapy, PRRT, or adjuvant systemic therapy following completed surgery; preoperative or neoadjuvant systemic therapy, if no subsequent surgery is already planned, is also classified as Systemic Therapy.
**9. Localized Therapy**	Local tumor-directed interventions, e.g., radiotherapy, SBRT, RFA, MWA, TACE or SIRT.

Supplementary Table 10. *Therapeutic categories and classification criteria. This table summarizes the nine therapeutic categories used to classify tumor board and LLM-generated recommendations. Each category is defined by the type of intervention or management strategy and key criteria for classification. Abbreviations: SBRT, stereotactic body radiotherapy; RFA, radiofrequency ablation; MWA, microwave ablation; TACE, transarterial chemoembolization; SIRT, selective internal radiation therapy.*
